# Distinct dynamics of parental 5-hydroxymethylcytosine during human preimplantation development regulate early lineage gene expression

**DOI:** 10.1038/s41556-024-01475-y

**Published:** 2024-07-30

**Authors:** Dan Liang, Rui Yan, Xin Long, Dongmei Ji, Bing Song, Mengyao Wang, Fan Zhang, Xin Cheng, Fengyuan Sun, Ran Zhu, Xinling Hou, Tianjuan Wang, Weiwei Zou, Ying Zhang, Zhixin Pu, Jing Zhang, Zhiguo Zhang, Yajing Liu, Yuqiong Hu, Xiaojin He, Yunxia Cao, Fan Guo

**Affiliations:** 1 Department of Obstetrics and Gynecology, The First Affiliated Hospital of Anhui Medical University, Hefei, China; 2 Key Laboratory of Organ Regeneration and Reconstruction, State Key Laboratory of Stem Cell and Reproductive Biology, Institute for Stem Cell and Regeneration, Institute of Zoology, University of Chinese Academy of Sciences, Chinese Academy of Sciences, Beijing, China; 3 Beijing Institute for Stem Cell and Regenerative Medicine, Beijing, China; 4 NHC Key Laboratory of Study on Abnormal Gametes and Reproductive Tract, Hefei, China; 5 Reproductive Medicine Center, Department of Obstetrics and Gynecology, Shanghai General Hospital, Shanghai Jiao Tong University School of Medicine, Shanghai, China

**Keywords:** Developmental biology, Epigenetics

## Abstract

The conversion of DNA 5-methylcytosine (5mC) to 5-hydroxymethylcytosine (5hmC) by TET enzymes represents a significant epigenetic modification, yet its role in early human embryos remains largely unknown. Here we showed that the early human embryo inherited a significant amount of 5hmCs from an oocyte, which unexpectedly underwent de novo hydroxymethylation during its growth. Furthermore, the generation of 5hmC in the paternal genome after fertilization roughly followed the maternal pattern, which was linked to DNA methylation dynamics and regions of sustained methylation. The 5hmCs persisted until the eight-cell stage and exhibited high enrichment at OTX2 binding sites, whereas knockdown of OTX2 in human embryos compromised the expression of early lineage genes. Specifically, the depletion of 5hmC affected the activation of embryonic genes, which was further evaluated by ectopically expressing mouse Tet3 in human early embryos. These findings revealed distinct dynamics of 5hmC and unravelled its multifaceted functions in early human embryonic development.

## Abstract (web summary)

Liang, Yan, Long, Ji et al. find that the origin and dynamics of 5-hydroxymethylcytosine (5hmC) during early development are not conserved between humans and mice and that 5hmC contributes to the activation of human embryonic genes.

## Main

Epigenetic regulation is critical for human early embryo development, yet the dynamics of the epigenome and its relevance at the beginning of human life remain elusive. DNA methylation (5mC), which is dominant in CpG dinucleotides in mammals, can be faithfully inherited by daughter cells during mitosis and serves as a regulatory mark for chromatin binding proteins and modifiers to influence gene expression and chromosome structure^1–3^. This comparatively static landscape is perturbed locally by the TET (ten-eleven-translocation) family of dioxygenases, by which 5mC is converted into sequential oxidative derivates such as 5-hydroxymethylcytosine (5hmC), 5-formylcytosine (5fC) and 5-carboxylcytosine (5caC)^4–6^. The latter two are recognized and excised by thymine DNA glycosylase (TDG) through the base excision repair (BER) pathway to induce DNA demethylation in embryonic stem (ES) cells and somatic tissues^7–9^. Although representing only a tiny proportion of all 5mC, 5hmC is still much more abundant than 5fC and 5caC in somatic cells, suggesting that it may have additional biological functions beyond its role as an intermediate in DNA demethylation^10–12^.

In marked contrast to the case in somatic cells, the epigenome shows rapid turnover in mammalian zygotes, in which DNA methylation reprogramming is a landmark event and is conserved between humans and mice^13–16^. The erasure of DNA methylation in mouse zygotes involves Tet3-mediated 5hmC generation^17^ and has been comprehensively studied by base-resolution mapping of 5hmC during mouse embryogenesis^18^. Notably, the zygotic 5hmC is preferentially enriched in the paternal but not in maternal genome^18^ and its loss has limited effects on zygotic genome activation (ZGA) and preimplantation development in mice^17,19,20^, prompting further exploration of biological significance of 5hmC in human early embryos. In this study, we set out to dissect 5hmC in human gametes, preimplantation embryos and ES cells, and attempt to unveil the dynamics and functions of 5hmC in early human development.

## Results

### Whole-genome sequencing of 5hmC in early human embryos

**Extended Data Fig. 1:**
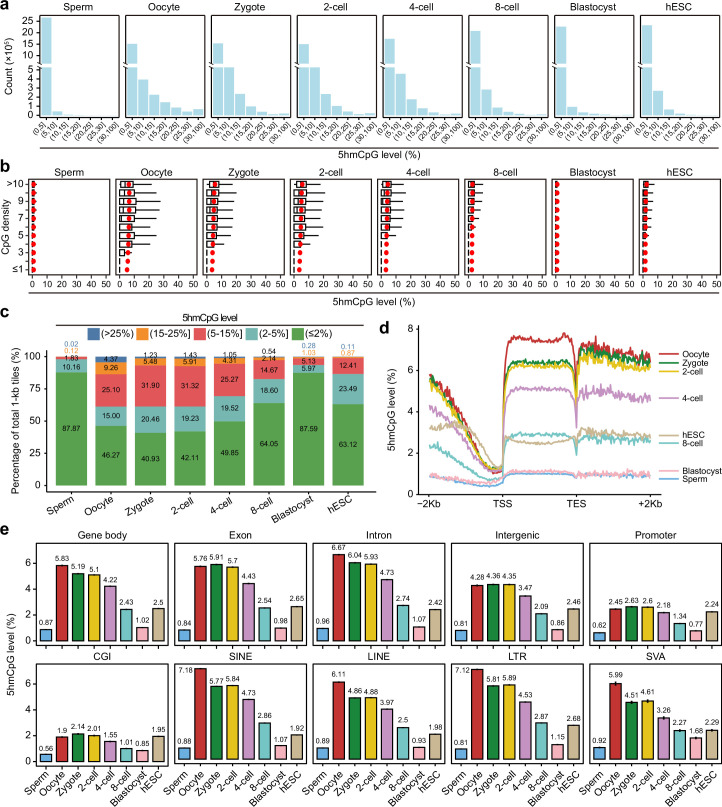
The distribution of 5hmCpGs in the genome of human gametes, early embryos and ES cells. **a**, Number of 1-kb tiles grouped by 5hmCpG levels in human gametes, preimplantation embryos and hESC. **b**, 5hmCpG levels of 1-kb tiles grouped by CpG density. Aggregated biological replicates were used. Sperm, n = 2; oocyte, n = 4; zygote, n = 3; 2-cell, n = 3; 4-cell, n = 4; 8-cell, n = 5; blastocyst, n = 3; hESC, n = 1. Red dots indicate the average 5hmCpG level. **c**, Proportions of 1-kb tiles with ≤ 2%, 2–5%, 5–15%, 15–25% and >25% 5hmCpG levels across the human genome (hg19). **d**, Average 5hmCpG levels on the gene body and flanking regions (±2 kb) in human gametes, preimplantation embryos and hESC. TSS, transcription start site; TES, transcription end site. **e**, 5hmCpG levels on representative genomic elements and regions in human gametes, preimplantation embryos and hESC. Error bar, mean ± se. Aggregated biological replicates were used. Sperm, n = 2; oocyte, n = 4; zygote, n = 3; 2-cell, n = 3; 4-cell, n = 4; 8-cell, n = 5; blastocyst, n = 3; hESC, n = 1. CGI, CpG island.

To study the role of 5hmC in early human development, we utilized an optimized APOBEC-coupled DNA sequencing method^18,21^ to generate whole-genome DNA hydroxymethylation maps of human gametes, preimplantation embryos and ES cells (Supplementary Table 1). On average, 45,768,077 CpG dyads and 832,333,955 CH sites were captured in each cell type (Supplementary Table 1). In general, the 5hmCpG landscape showed an extensively low but CpG-density-independent distribution across all stages investigated (Extended Data Fig. 1a,b), with the exceptions that oocytes, zygotes, two-cell and four-cell embryos had a prominent proportion of high-5hmCpG 1-kb tiles (15–25% level) (Extended Data Fig. 1a–c).

### Unique dynamics of 5hmC in human oocytes and early embryos

**Fig. 1:**
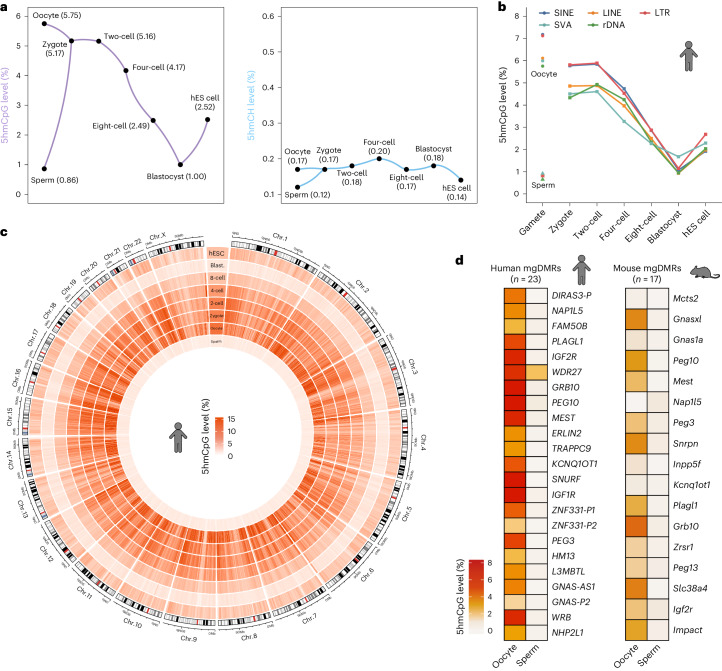
Landscape of 5hmC in human gametes and early embryos. **a**, Dynamics of 5hmCpG and 5hmCH levels in human gametes, preimplantation embryos and hES cells. **b**, Dynamics of 5hmCpG levels at SINEs, LINEs, LTRs, SVAs and rDNA in human gametes, preimplantation embryos and hES cells. **c**, Circos plot showing genome-wide 5hmCpG levels in human gametes, preimplantation embryos and hES cells. Average 5hmCpG levels in 1-Mb tiles were calculated across different samples. **d**, 5hmCpG levels at mat igDMRs (mgDMRs) in human and mouse gametes. The mouse dataset was retrieved from GSE186357. Biological replicates were merged. Sperm, *n* = 2; oocyte, *n* = 4; zygote, *n* = 3; two-cell, *n* = 3; four-cell, *n* = 4; eight-cell, *n* = 5; blastocyst, *n* = 3; hES cells, *n* = 1. LINE, long interspersed nuclear element; LTR, long terminal repeat; SVA, SINE-VNTR-Alus; rDNA, ribosomal DNA.

We next analysed the dynamics of 5hmC across early human development. Unexpectedly, human oocytes had a 3.6-fold higher global 5hmCpG level (5.75%) than that in mice (Fig. 1a and Extended Data Fig. 1d). This comparable level of 5hmCpG was also detected in zygotes (5.17%), suggesting that either the paternal genome undergoes 5mCpG oxidation or the maternal genome continues to generate additional 5hmCpG upon fertilization. The level of 5hmCpG persisted to the two-cell stage and then decreased at each stage in later cleavage embryos, reaching the lowest level in blastocysts (Fig. 1a and Extended Data Fig. 1d). Unlike that of 5hmCpG, the 5hmC level in the CH context was relatively stable between gametes and early embryos, ranging between 0.12% and 0.20%, in accordance with the fact that 5hmC is predominantly produced in CpG dinucleotides (Fig. 1a). We thus exclusively examined 5hmC in CpGs in the downstream results.

**Extended Data Fig. 2:**
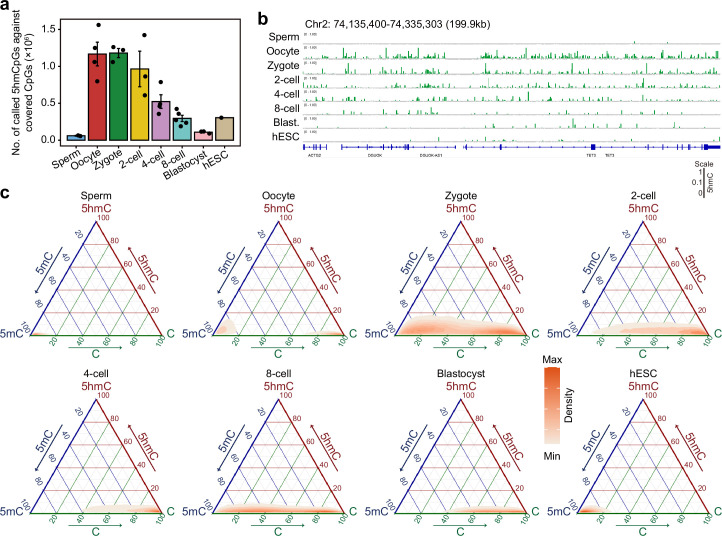
Dynamic of 5hmCpG sites in human preimplantation embryos and hESCs. **a**, Number of significant 5hmCpGs against covered CpGs in human gametes, preimplantation embryos and hESCs. Error bar, mean ± se. **b**, Representative 5hmCpG loci in human gametes, preimplantation embryos and hESC. **c**, Ternary plots showing the levels of unmodified cytosines, 5mC and 5hmC on 1-kb tiles across the human genome (hg19) in human gametes, preimplantation embryos and hESC.

The dynamics of 5hmC in different genomic elements generally mimicked the global trend (Extended Data Fig. 1e), with the interesting observation that repetitive sequences contained relatively higher levels of 5hmC beginning at the oocyte stage (Fig. 1b and Extended Data Fig. 1e). Nevertheless, the pattern of 5hmC distribution was indistinguishable among different chromosomes across cell types (Fig. 1c). The dynamics of 5hmC were further validated by identifying statistically significant 5hmC sites, which displayed a pattern analogous to that shown in Fig. 1a (Extended Data Fig. 2a,b). Additionally, the ternary plot of C, 5mC and 5hmC in 1-kb tiles revealed more details related to the 5hmC dynamics (Extended Data Fig. 2c). Compared with mouse oocytes, maternal imprinted germline differentially methylated regions (mat igDMRs) exhibited much more abundant 5hmC in humans (Fig. 1d and Supplementary Table 2), coinciding with the establishment of DNA methylation at these loci during human oocyte growth^22^.

### Growing human oocytes undergo de novo 5hmC generation

**Fig. 2:**
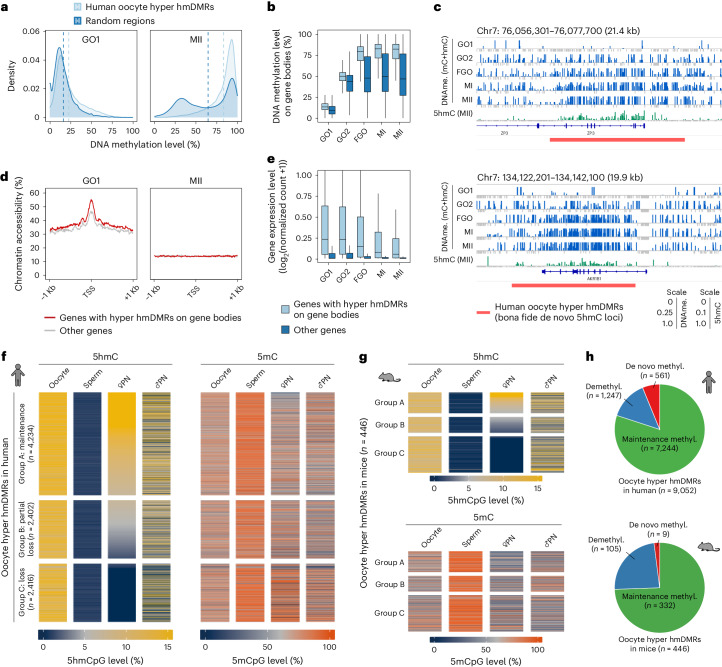
De novo 5hmC generated during human oogenesis and inherited after fertilization. **a**, DNA methylation levels of human oocyte hyper hmDMRs and random selected regions in growing oocyte 1 (GO1) and MII oocytes (MII). Dashed lines indicate the median level. **b**, DNA methylation levels within gene bodies during oogenesis. Genes were divided into two groups according to whether their gene bodies overlapped with oocyte hyper hmDMRs. Single-cell replicates were merged. GO1, *n* = 49; GO2, *n* = 37; FGO, *n* = 81; MI, *n* = 155; MII, *n* = 90. FGO, fully grown oocyte; MI, oocyte in metaphase I. **c**, DNA methylation and 5hmCpG levels at representative human oocyte hyper hmDMRs in which 5hmC were de novo generated during oogenesis. **d**, Chromatin accessibility of genes with hyper hmDMRs within gene bodies in GO1 and MII. **e**, Expression levels of genes with hyper hmDMRs within gene bodies. Single-cell chromatin accessibility, DNA methylation and expression datasets of human growing and mature oocytes were retrieved from GSE154762. hmDMRs, hydroxymethylated differentially methylated regions; DNAme., DNA methylation; TSS, transcription start site. **f**,**g**, Dynamics of 5hmCpG and 5mCpG levels in human and mouse oocyte hyper hmDMRs during fertilization. Groups A–C were divided according to the 5hmCpG level in human maternal genomes of zygotes or mouse female pronuclei. Biological replicates were merged. The DNA methylation dataset for human gametes and preimplantation embryos was retrieved from GSE81233. The mouse dataset was retrieved from GSE186357. ♀PN, female pronucleus; ♂PN, male pronucleus. **h**, Proportions of regions exhibiting de novo methylation (De novo methyl.), demethylation (Demethyl.) and regions with stable methylation (Maintenance methyl.) in human and mouse oocyte hyper hmDMRs upon fertilization.

**Extended Data Fig. 3:**
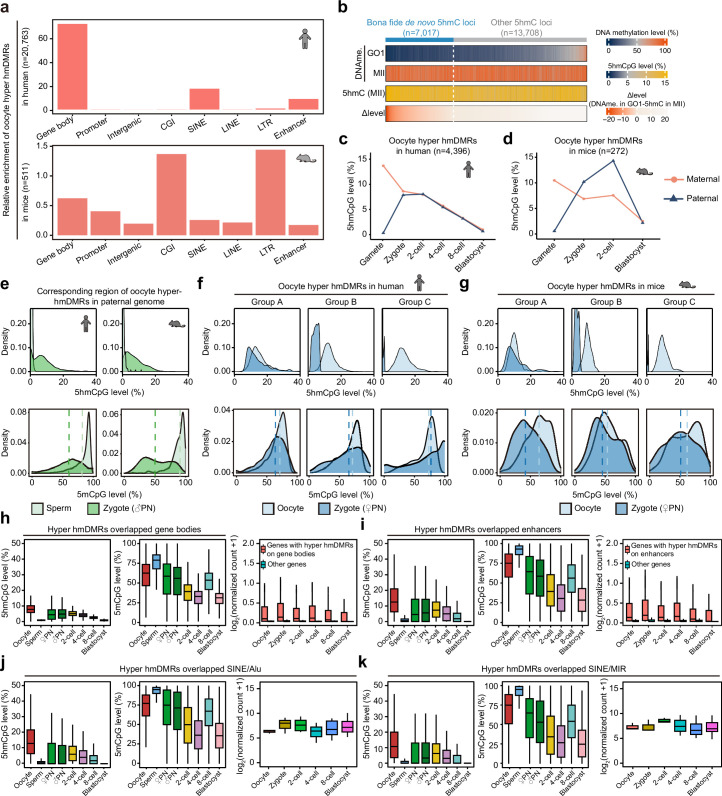
The characteristics of 5hmC inherited from human oocytes. **a**, Enrichment of oocyte hyper hmDMRs on representative genomic elements and regions in humans and mice. **b**, Levels of DNA methylation (DNAme.) and 5hmCpG on human oocyte hyper hmDMRs. Bona fide de novo 5hmC loci were regions with Δlevel < 0. **c,d**, Dynamics of parental-specific 5hmCpG levels on oocyte hyper hmDMRs in human (**c**) and mouse (**d**) gametes and preimplantation embryos. **e–g**, 5hmCpG and 5mCpG levels in corresponding regions of oocyte hyper hmDMRs in the paternal genome **(e)** and oocyte hyper hmDMRs in the human **(f)** and mouse **(g)** maternal genomes during fertilization. Dashed lines indicate median levels. Groups A-C are defined in Fig. 2f,g. **h–k**, 5hmCpG levels, 5mCpG levels and corresponding gene expression levels in representative genomic regions overlapping with oocyte hyper hmDMRs. For 5hmC, aggregated biological replicates were used. Sperm, n = 2; oocyte, n = 4; zygote, n = 3; 2-cell, n = 3; 4-cell, n = 4; 8-cell, n = 5; blastocyst, n = 3. The DNA methylation dataset for human preimplantation embryos was retrieved from GSE81233. Aggregated biological replicates were used. Sperm, n = 21; oocyte, n = 32; female pronuclei, n = 12; male pronuclei, n = 13; 2-cell, n = 10; 4-cell, n = 15; 8-cell, n = 10; blastocyst, n = 11. The expression dataset for human preimplantation embryos was retrieved from GSE36552. The number of biological replicates in each stage were: oocyte, n = 3; zygote, n = 3; 2-cell, n = 6; 4-cell, n = 12; 8-cell, n = 20; blastocyst, n = 30.

To further dissect the origin and fate of 5hmC in human MII (metaphase II) oocytes, we first identified 20,763 hydroxymethylated (hm)DMRs compared with sperm (Extended Data Fig. 3a and Supplementary Table 3). These hmDMRs had a median length of 7,600 bp and a median 5hmC level of 13.69%. Notably, this number was almost 40-fold higher than that called in mouse metaphase II (MII) oocytes by the same criteria (*n* = 511, 1,900 bp median length and 10.03% median level). Additionally, the hmDMRs were highly enriched in gene bodies, and some of them also overlapped locally with known human cell enhancers and short interspersed nuclear elements (SINEs), the distribution of which was distinct from that in mice (Extended Data Fig. 3a). We next incorporated our published single-cell multi-omics data of human growing and mature oocytes^22^ for detailed analysis. Those oocyte hmDMRs and their located gene bodies were de novo methylated during oogenesis and represented significantly high DNA methylation levels (88.30% and 82.30% in median level), which is in contrast to the bimodal distribution of DNA methylation of the remaining loci (Fig. 2a,b). A total of 39.49% of oocyte hyper hmDMRs were low in DNA methylation level (<0.15) in the initial growing stage (GO1 stage), demonstrating that de novo DNA hydroxymethylation indeed occurred during human oocyte growth (Fig. 2c and Extended Data Fig. 3b). Moreover, those genes with oocyte hyper hmDMRs had promoters more accessible and were actively transcribed during human oogenesis (Fig. 2d,e), suggesting that both chromatin accessibility and transcription, together with TET protein activity, were involved in de novo DNA hydroxymethylation in human oocytes.

The fate of oocyte hyper hmDMRs after fertilization was further examined by distinguishing parental single nucleotide polymorphisms (Extended Data Fig. 3c,d). We defined loci with maintenance, partial loss and loss of their 5hmC levels between the oocyte and zygote stages (Fig. 2f,g). A total of 46.77% of human oocyte hyper hmDMRs exhibited maintained 5hmC levels in zygotes and two-cell embryos but gradually decreased afterwards (Fig. 2f and Extended Data Fig. 3c). Notably, the paternal genomes of both human and mouse zygotes generated significant levels of 5hmC in the regions corresponding to oocyte hyper hmDMRs (Fig. 2f,g and Extended Data Fig. 3c,d) and underwent DNA demethylation (Fig. 2f,g and Extended Data Fig. 3e), but the dynamics were different between the two species (Extended Data Fig. 3c,d). In addition, 80.0% of human oocyte hyper hmDMRs exhibited relatively stable 5mC levels, whereas 13.8% and 6.2% were subjected to demethylation or de novo methylation in the maternal genome, respectively, at the zygote stage (Fig. 2f–h and Extended Data Fig. 3f,g). The functional relevance of 5hmC inherited from oocyte hyper hmDMRs was then evaluated in early human embryos. Despite DNA demethylation, the genes with maternally inherited hyper hmDMRs in either the gene body or enhancer exhibited higher transcriptional level compared with other genes (Extended Data Fig. 3h,i) and the SINE repeat sequences were similar (Extended Data Fig. 3j,k). These results revealed that the majority of human oocyte-derived 5hmC could be transmitted and maintained to as late as the two-cell stage and may be functionally linked with the corresponding gene and repeat expression in early embryos.

### The paternal genome generates 5hmC upon fertilization

**Fig. 3:**
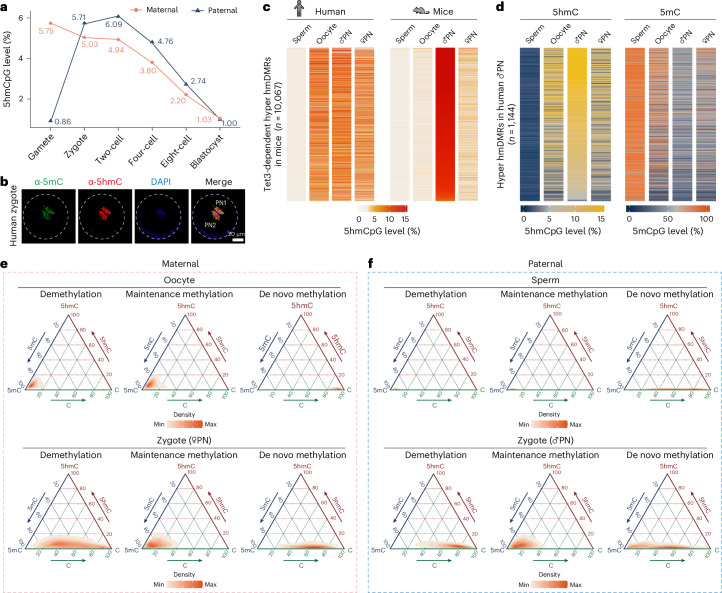
Tracking 5hmC dynamics from parental genomes in human preimplantation embryos. **a**, Dynamics of parental-specific 5hmCpG levels in human gametes and preimplantation embryos. **b**, 5mC and 5hmC co-staining of human zygotes (20 h after intracytoplasmic sperm injection). The dashed lines mark the boundary of the zygote. A representative image from five human zygotes is shown. **c**, Dynamics of parental-specific 5hmCpG levels on Tet3-dependent hyper hmDMRs identified in mice. These hyper hmDMRs in mouse were identified in our previously published study^18^. **d**, Dynamics of parental-specific 5hmCpG and 5mCpG levels on hyper hmDMRs in human paternal genome of zygotes during fertilization. **e**,**f**, Ternary plots showing the levels of unmodified cytosines, 5mC and 5hmC in parental-specific regions exhibiting demethylation, de novo methylation and regions with stable (maintenance) methylation during fertilization.

**Extended Data Fig. 4:**
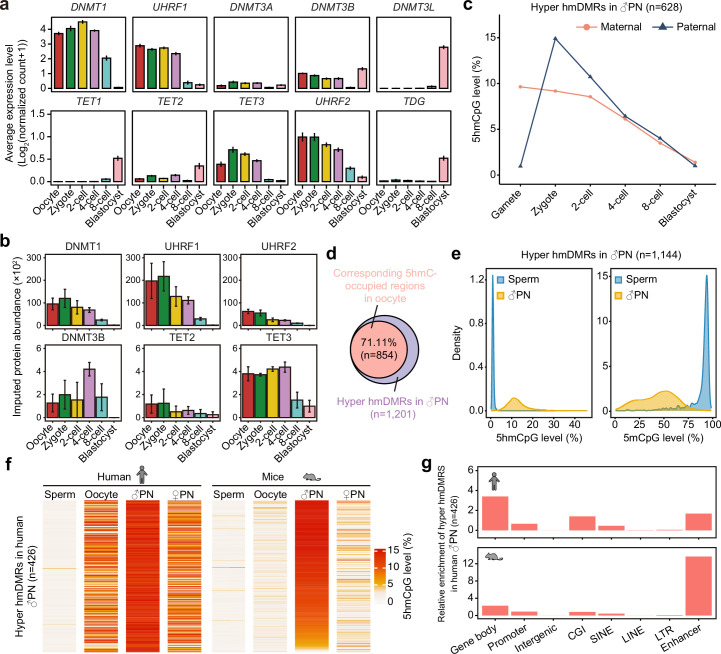
The pattern of 5hmC generated in the paternal genome after fertilization. **a**, Expression levels of representative genes involved in DNA (de)methylation in human preimplantation embryos. Error bar, mean ± se. The gene expression dataset for human preimplantation embryos was retrieved from GSE36552. The number of biological replicates in each stage were: oocyte, n = 3; zygote, n = 3; 2-cell, n = 6; 4-cell, n = 12; 8-cell, n = 20; blastocyst, n = 30. **b**, Expression levels of representative proteins involved in DNA (de)methylation in human preimplantation embryos. Error bar, mean ± se. The protein data for human preimplantation embryos was retrieved from PXD024267. The number of biological replicates in each stage were: oocyte, n = 5; zygote, n = 5; 2-cell, n = 4; 4-cell, n = 13; 8-cell, n = 9; blastocyst, n = 10. **c**, Dynamic of parental-specific 5hmCpG levels on hyper hmDMRs in paternal genome of zygotes across human gametes and preimplantation embryos. **d**, Percentage of hyper hmDMRs in paternal genome of zygotes that were also 5hmC-occupied at corresponding regions in oocytes. **e**, 5hmCpG and 5mCpG levels at hyper hmDMRs in paternal genome of zygotes. **f**, Comparing 5hmCpG levels at human paternal hyper hmDMRs and homologous regions in mice. **g**, Genome distribution of human paternal hyper hmDMRs at homologous regions in the human and mouse genomes. Source data

Next, the global dynamics of 5hmC in the paternal genome were deduced. In contrast to the maternal genome, which inherited 5hmC from oocytes, the paternal genome underwent a sharp increase in global 5hmC level upon fertilization (5.71%) that persisted to the two-cell stage (6.09%) (Fig. 3a). Both parental genomes showed a gradual decrease in 5hmC in late-stage cleavage embryos and reached comparable levels in blastocysts (Fig. 3a). The presence of 5hmC signals in both human parental pronucleus was further confirmed by immunostaining (Fig. 3b), which was different from mouse zygote as previously reported^23,24^. Because TET3 was the most abundant in mRNA and protein level than the other two TET members in human oocytes and zygotes (Extended Data Fig. 4a,b), and its orthologous gene in mice is responsible for 5hmC production upon fertilization^18^, we first examined whether the Tet3-targeted loci (*n* = 10,067) identified in mice would be conservatively modified in humans (Fig. 3c). Those loci were also generally modified with 5hmC in the paternal genomes of human zygotes, albeit at lower levels than in mice (Fig. 3c). Also, in contrast to the case in mice, the human maternal genome was targeted beginning in oocytes and maintained in zygotes (Fig. 3c). We then identified 1,144 paternal hyper hmDMRs (7,600 bp in median length and 11.82% in median level) in zygotes compared with sperm, which showed rapid turnover from the two-cell stage onwards (Fig. 3d, Extended Data Fig. 4c and Supplementary Table 4). Although both the oocyte and paternal genomes of human zygotes shared significant levels of 5hmC at paternal hyper hmDMRs (Fig. 3d and Extended Data Fig. 4d), there was a difference in that the paternal genome underwent dramatic DNA demethylation (Fig. 3d and Extended Data Fig. 4e). To further examine that 5mC oxidation in human zygotes may also require TET3, 426 human paternal hyper hmDMRs were evaluated in mice and these loci were highly hydroxymethylated in both species (Extended Data Fig. 4f,g). These results suggested that TET protein targeted to the conservative genomic loci in the paternal genome of both mouse and human zygotes.

### The relationship between 5hmC and 5mC in human zygotes

**Extended Data Fig. 5:**
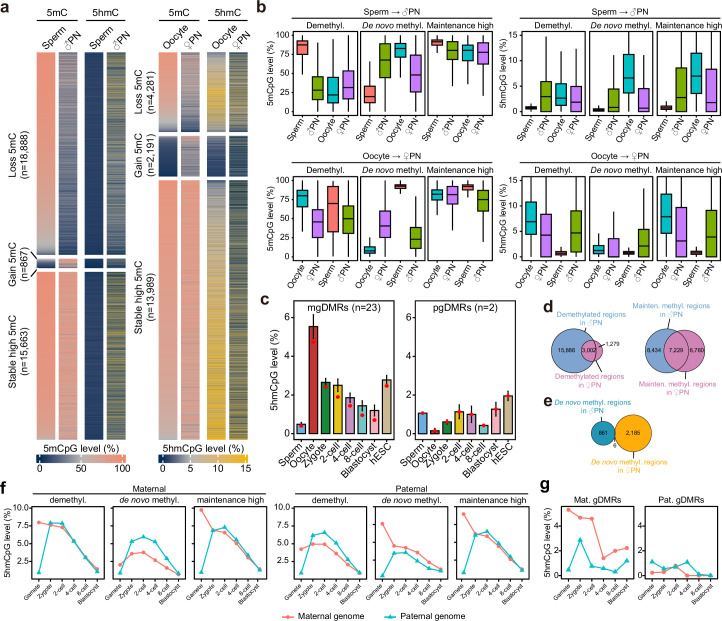
The relationship between 5hmC and 5mC in humans upon fertilization. **a,b**, Effects of 5mCpG and 5hmCpG on the 5mC loss (Demethyl.), 5mC gain (de novo methyl.) and stable high 5mC (Maintenance high) regions in sperm and paternal genome of zygotes, oocytes and maternal genome of zygotes. Aggregated biological replicates were used. Sperm, n = 2; Oocyte, n = 4; Zygote, n = 3. **c**, 5hmCpG levels at maternal and paternal igDMRs (23 regions for mgDMRs and 2 regions for pgDMRs) in human gametes, preimplantation embryos and hESC. Aggregated biological replicates were used. Sperm, n = 2; Oocyte, n = 4; Zygote, n = 3; 2-cell, n = 3; 4-cell, n = 4; 8-cell, n = 5; Blastocyst, n = 3; hESC, n = 1. Data are presented as mean values ± SEM. Red dots indicate the average 5hmCpG level. **d,e**, Overlap of DNA demethylation regions (**d, left**), maintenance high methylation regions (**d, right**), and de novo methylation regions (**e**) in paternal and maternal genome of zygotes. **f,g**, Allele-specific 5hmCpG dynamics in parental demethylation, de novo methylation, maintenance high methylation regions (**f**) and germline differentially methylated regions (gDMRs) (**g**) across human preimplantation development at single CpG level. Source data

Unlike in mice, in which loci that were demethylated in the parental genomes of zygotes were abundantly enriched in 5hmC^18^, the maternal genomes of human zygotes possessed noticeable levels of 5hmC in regions with maintained DNA methylation (Fig. 2 and Extended Data Fig. 3). We thus further explored the relationship between 5mC and 5hmC in the parental genomes upon fertilization. A total of 18,888, 15,663 and 867 loci exhibited DNA demethylation (median length of 20,200 bp), maintenance (median length of 10,900 bp) or de novo methylation (median length of 2,200 bp) in the paternal genome, respectively (Extended Data Fig. 5a,b). And 4,281, 2,191 and 13,989 loci were identified as DNA demethylation (median length of 7,900 bp), de novo (median length of 7,300 bp) or maintenance methylation (median length of 11,500 bp) in the maternal genome, respectively. As expected, 5hmC was coupled with DNA demethylation in genomes from both parents in human zygotes (Fig. 3e,f and Extended Data Fig. 5a,b). The paternal genome of human zygotes, unlike the maternal genome, underwent de novo generation without inheriting 5hmC from sperm in loci where DNA methylation was maintained (Fig. 3e,f and Extended Data Fig. 5a,b), exemplified by parental igDMRs (Extended Data Fig. 5c). Although de novo DNA methylation occurred to a mild extent in human zygotes, these loci indeed generated significant 5hmC, which is very different from the case in mice^18^ (Fig. 3e,f and Extended Data Fig. 5a,b). Both DNA demethylated loci and loci with maintained methylation overlapped substantially between parental genomes (Extended Data Fig. 5d), suggesting a common mechanism for targeting these loci to execute 5mC oxidation. Notably, those de novo DNA methylation loci showed either gain or loss of 5mC levels in only one parental genome (Extended Data Fig. 5b,e). The dynamics of 5hmC at CpG sites were further evaluated throughout developmental stages (Extended Data Fig. 5f,g). This revealed a noteworthy observation that both parental genomes generated 5hmC after fertilization at the corresponding regions of maternal de novo DNA methylation loci, which was distinct from the maternal genome inheriting 5hmC from the oocyte at DNA demethylation and maintenance methylation loci, such as mat igDMRs (Extended Data Fig. 5f,g).

### Gene body hydroxymethylation correlates with gene expression

**Extended Data Fig. 6:**
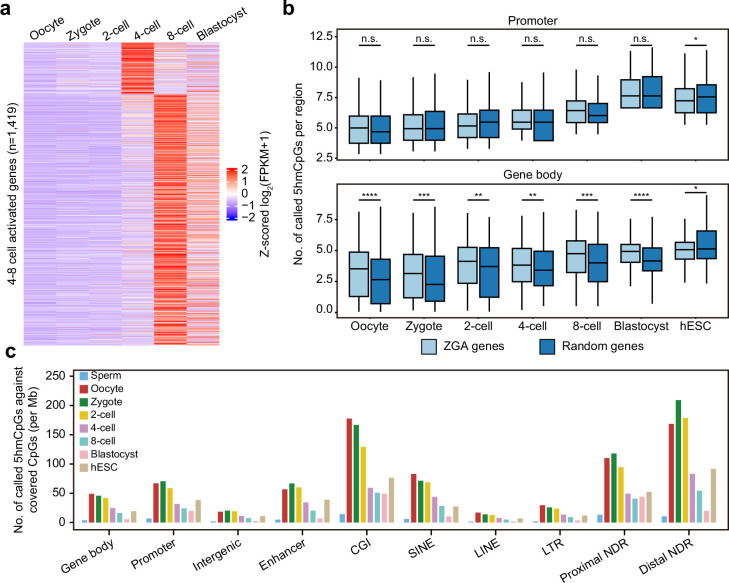
The correlation of 5hmC with corresponding gene expression in human early embryos. **a**, Expression levels of 4–8 cell activated genes (ZGA genes) in human oocytes and preimplantation embryos. The expression dataset for human oocytes and preimplantation embryos was retrieved from GSE197265. There are two biological replicates in each stage. **b**, Number of significant 5hmCpGs within the promoter or gene body of ZGA genes and random selected gene in human oocytes, preimplantation embryos and hESCs. ZGA, zygotic genome activation. Aggregated biological replicates were used. Oocyte, n = 4; Zygote, n = 3; 2-cell, n = 3; 4-cell, n = 4; 8-cell, n = 5; Blastocyst, n = 3; hESC, n = 1. P-values were calculated by the two-sided Wilcoxon signed-rank test. **c**, Number of significant 5hmCpGs against covered CpGs on representative genomic elements and regions in human gametes, preimplantation embryos and hESC. Mb, megabase; NDR, nucleosome depleted region.

We hypothesized that 5hmC might have activities in early human embryos other than participating in 5mC turnover, as highlighted by oocyte hyper hmDMRs, which were associated with active gene expression (Extended Data Fig. 3). To address this question, we first explored whether 5hmC was a distinguishable marker for human embryonic activation genes. ZGA in humans primarily occurs between the four-cell and eight-cell stages (Extended Data Fig. 6a). We did not observe any significant difference in 5hmC deposition in promoters between ZGA genes and other genes throughout the whole developmental period (Extended Data Fig. 6b). However, within the gene body regions, we consistently observed a higher number of high-confidence 5hmC sites on ZGA genes compared with random genes throughout human preimplantation embryonic development—a pattern not observed at promoter regions (Extended Data Fig. 6b), indicating that 5hmC enrichment in the gene body may serve as a marker for late-stage ZGA gene expression in human embryos.

### 5hmCs are linked with active enhancers and genes expression

**Fig. 4:**
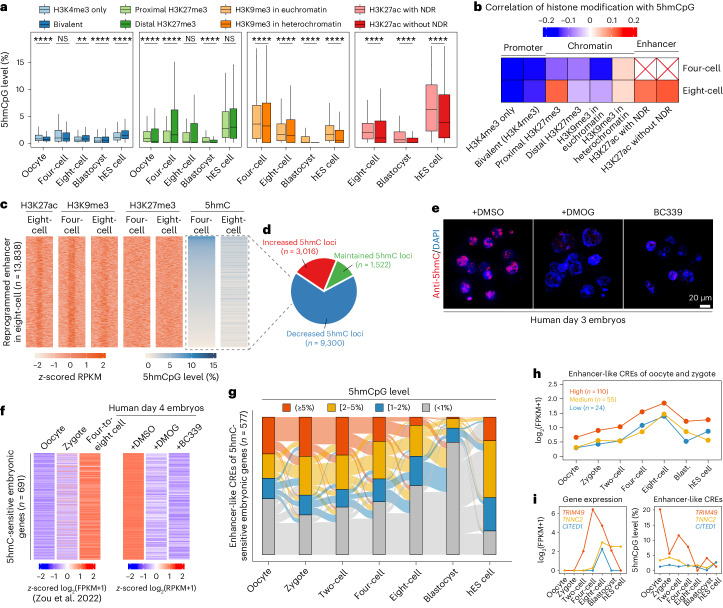
The link between 5hmC, histone modifications and genes activation in human early embryos. **a**, 5hmCpG levels at histone modification peaks identified in ChIP-seq. Aggregated biological replicates were used. Oocyte, *n* = 4; four-cell, *n* = 4; eight-cell, *n* = 5; blastocyst, *n* = 3; hES cells, *n* = 1. *P* values were calculated by the two-sided Wilcoxon signed-rank test. *P < 0.05; **P < 0.01; ***P < 0.001; ****P < 0.0001; NS, not significant. **b**, Correlations between 5hmCpG levels and histone modification signals (RPKM, reads per kilobase per million). Correlation was measured by Spearman’s rank correlation coefficient. **c**, 5hmCpG levels in four-cell and eight-cell embryos on human eight-cell reprogrammed enhancers. These enhancers were defined in ref. ^27^. **d**, Proportions of decreased (5hmCpG level in four-cell minus 5hmCpG level in eight-cell divided by 5hmCpG level in four-cell > 15%), increased (5hmCpG level in four-cell minus 5hmCpG level in eight-cell divided by 5hmCpG level in four-cell <−15%) and maintained 5hmC loci (|5hmCpG level in four-cell minus 5hmCpG level in eight-cell divided by 5hmCpG level in four-cell| ≤ 15%). **e**, 5hmC staining of control (DMSO-treated), DMOG- and BC339-treated human day 3 embryos. Representative images from three independent biological replicates are shown. **f**, Right: Heatmap showing the genes that were downregulated in both DMOG- and BC339-treated embryos. Left: These genes are activated in normal human early embryos at the 4–8 cell stage. For RNA-seq of DMSO-, DMOG- and BC339-treated human day 4 embryos, *n* = 21, 16 and 17, respectively. **g**, The Sankey plot showing the dynamics of 5hmC in enhancer-like CREs of 5hmC-sensitive embryonic genes across human early embryo development. **h**, Expression dynamic of 5hmC-sensitive embryonic genes with differential 5hmC levels at enhancer-like CREs in oocyte and zygote stage. High indicates that the 5hmC level is greater or equal to 5%; medium, greater or equal to 2%, less than 5%; and low: greater or equal to 1%, less than 2%. **i**, Left: Expression dynamics of representative genes with high, medium or low 5hmC levels at enhancer-like CREs. Right: 5hmC levels at enhancer-like CREs of these representative genes. FPKM, fragments per kilobase per million.

*Cis*-regulatory elements (CREs) are primed and marked with open chromatin in early human embryos and play roles in modulating gene transcription^25^. Notably, 5hm CpG sites also showed enrichment at proximal and distal nucleosome-depleted regions (NDRs) in early human embryos (Extended Data Fig. 6c). Additionally, the levels of 5hmC in active (H3K4me3-marked), repressive (H3K27me3-marked) or bivalent (marked with both) promoters were generally low in early human embryos (Fig. 4a). However, repressive H3K9me3-marked regions and H3K27me3-distal regions showed significant levels of 5hmC (Fig. 4a), coinciding with the erasure of these two histone marks in the four-cell and eight-cell stages^26–28^. Notably, H3K27ac-marked CREs and active enhancers (co-marked by H3K27ac and NDRs) possessed relatively high levels of 5hmC (Fig. 4a). And there was a positive correlation between the 5hmC and H3K27ac modifications in these two regions (Fig. 4b). To further investigate whether 5hmC was present before the enhancer became active or was generated after the active enhancer formed, we focused on a special cluster of enhancers that were co-marked with H3K27ac and H3K9me3 but not H3K27me3 and became active with the erasure of H3K9me3 between the four-cell and eight-cell stage in human embryos^27,28^. The results demonstrated the presence of 5hmC at these reprogrammed enhancers in four-cell embryos (Fig. 4c,d), and this mark partially persisted into eight-cell embryos, even when H3K9me3 was removed (Fig. 4c,d).

**Extended Data Fig. 7:**
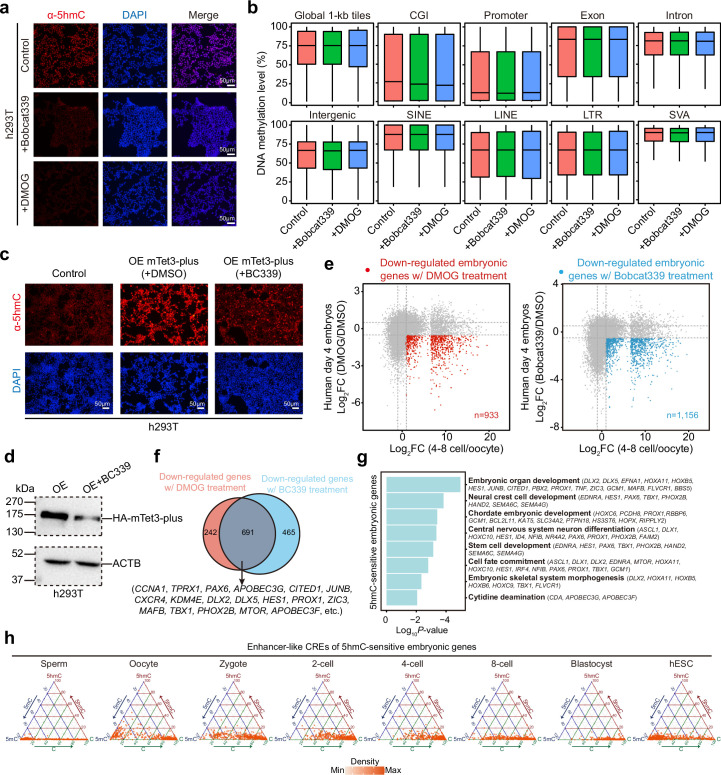
The reduction of global 5hmC level by small molecule inhibitors. **a**, 5hmC staining of control (DMSO-treated), Bobcat339- and DMOG- treated h293T cells. Representative images from two independent biological replicates were showed. **b**, DNA methylation levels of control (DMSO-treated), Bobcat339- and DMOG- treated h293T cells at genome-wide 1-kb tiles and different genomic elements. **c**, 5hmC staining of h293T cells without mTet3-plus transfection and Bobcat339 (BC339) treatment were used as negative control. mTet3-plus transiently transfected h293T cells were treated by 0.3% DMSO or 80 μM BC339 for 24 hrs to detect the 5hmC signals. OE, overexpression. Representative images from two independent biological replicates were showed. **d**, The abundance of HA-conjugated mouse enhanced Tet3 protein was detected by Western blotting in mTet3-plus overexpressed h293T cells with or without BC339 treatment, beta-actin (ACTB) was used as loading control. Representative images from two independent biological replicates were showed. **e**, Scatter plots comparing mRNA changes between DMOG- (n = 16) or Bobcat339- treated (n = 17) embryos and control embryos (n = 21) (y-axis), and mRNA changes from MII oocyte to 4–8 cell stage (x-axis) are shown. Embryonic genes are 4–8 cell activated genes. **f**, Overlap of down-regulated embryonic genes in human day 4 embryos with DMOG- or Bobcat339- treatment. **g**, Representative Gene Ontology (GO) terms for 5hmC-sensitive embryonic genes. P-value were calculated by Metascape. **h**, Ternary plots showing the levels of unmodified cytosines, 5mC and 5hmC in enhancer-like cis-regulatory elements (CREs) of 5hmC-sensitive embryonic genes in human gametes, early embryos and hESC. Enhancer-like CREs were co-marked by H3K27ac and NDR in human 8-cell embryos. Source data

To further investigate the role of 5hmC in transcriptional regulation, the dimethyloxalylglycine (DMOG) and Bobcat339 (BC339) were introduced to reduce the global 5hmC level in human early embryos. The performance of these two inhibitors was first verified in h293T cells, representing reduced global 5hmC signals but undisturbed 5mC level across genome (Extended Data Fig. 7a,b), as previously reported^29,30^. Additionally, we have also observed that the presence of BC339 induces destabilization of TET3 protein (Extended Data Fig. 7c,d), which is consistent with the result in previous study^29^. The 5hmC signals of human day 3 embryos were expectedly decreased by treating with either DMOG or BC339 (Fig. 4e). RNA sequencing (RNA-seq) of single human embryos showed that there were 933 and 1,156 embryonic genes downregulated in DMOG- (*n* = 16) and BC339-treated (*n* = 17) groups respectively, compared with dimethylsulfoxide (DMSO)-treated (*n* = 21) embryos (Extended Data Fig. 7e). Moreover, 691 genes were downregulated in both DMOG- and BC339-treated embryos, suggesting a common on-target effect of 5hmC reduction (Extended Data Fig. 7f and Supplementary Table 5). These genes functioned in embryonic or stem cell development, or cell fate commitment (Fig. 4f and Extended Data Fig. 7g). Notably, 31.7% (219 out of 691) of downregulated genes were strongly activated in 4–8 cell human embryos (Extended Data Fig. 6a and Extended Data Fig. 7f), including *CCNA1*, *TPRX1*, *PAX6*, *APOBEC3G*, *CITED1*, *JUNB*, *CXCR4* and *KDM4E*. Among them, *CCNA1* was shown to function in regulating the maternal-to-zygote transition^31^. *TPRX1* was a marker of eight-cell-like cells in hES cells^32^. Additionally, significant proportions of the enhancer-like CREs (co-marked by H3K27ac and NDRs) of those 691 5hmC-sensitive embryonic genes did contain 5hmC modifications from the oocyte to the eight-cell embryo (Fig. 4g and Extended Data Fig. 7h). And the level of 5hmC at these CREs was generally correlated with corresponding gene expression, exemplified by *TRIM49*, *TNNC2* and *CITED1* (Fig. 4h,i). These results indicated that 5hmC was involved in ZGA gene expression of human early embryos.

### Ectopically generated 5hmC in human early embryos by mTet3

**Fig. 5:**
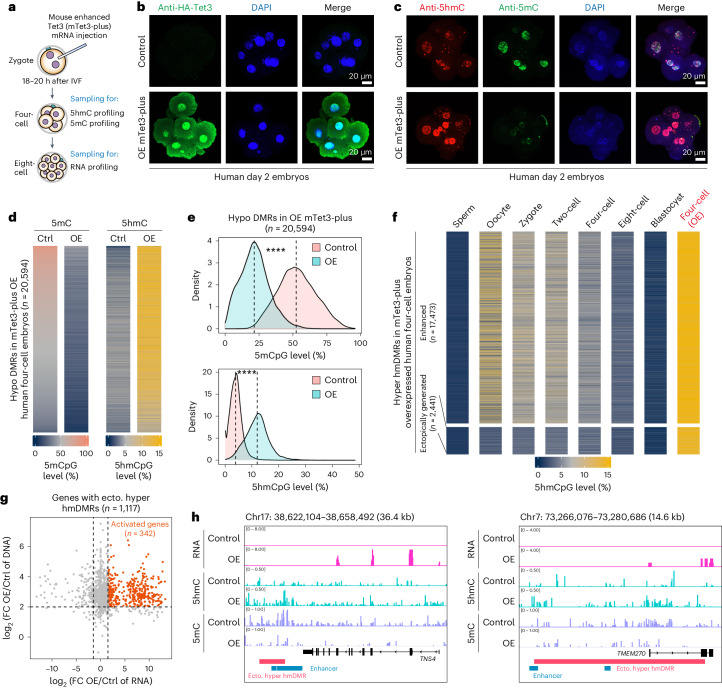
Analysing 5mC, 5hmC and gene activation in mTet3-plus-overexpressed human early embryos. **a**, Schematic illustration of the experimental approach. IVF, in vitro fertilization. **b**, HA staining was performed on human day 2 embryos, with control and mouse enhanced Tet3 (mTet3-plus) mRNA microinjection. The N terminus of mTet3-plus protein was tagged with HA. Representative images from five groups of control and mTet3-plus overexpressed (OE) human day 2 embryos were presented. **c**, 5hmC and 5mC co-staining of control and mTet3-plus mRNA microinjected human day 2 embryos. Representative images from nine groups of control and mTet3-plus overexpressed human day 2 embryos are shown. **d**, Heatmaps showing the 5mCpG and 5hmCpG levels on hypo DMRs identified in mTet3-plus- OE human four-cell embryos when compared with control embryos. Ctrl, control. **e**, Density plots showing the 5mCpG and 5hmCpG levels on hypo DMRs identified in mTet3-plus OE human four-cell embryos when compared with control embryos. Dashed lines indicate the median level. *P* values were calculated by a two-sided Wilcoxon signed-rank test. ****P < 2.2 × 10^−16^. **f**, Heatmap showing the dynamics of 5hmCpG levels on hyper hmDMRs of mTet3-plus OE human four-cell embryos during early embryo development. **g**, Scatter-plot comparing mRNA level changes of mTet3-plus OE and control human eight-cell embryos (*x* axis) and 5hmC/5mC changes of mTet3-plus OE and control human four-cell embryos (*y* axis). Ecto., ectopic; FC, fold change. **h**, Representative activated genes in mTet3-plus OE human eight-cell embryos.

**Extended Data Fig. 8:**
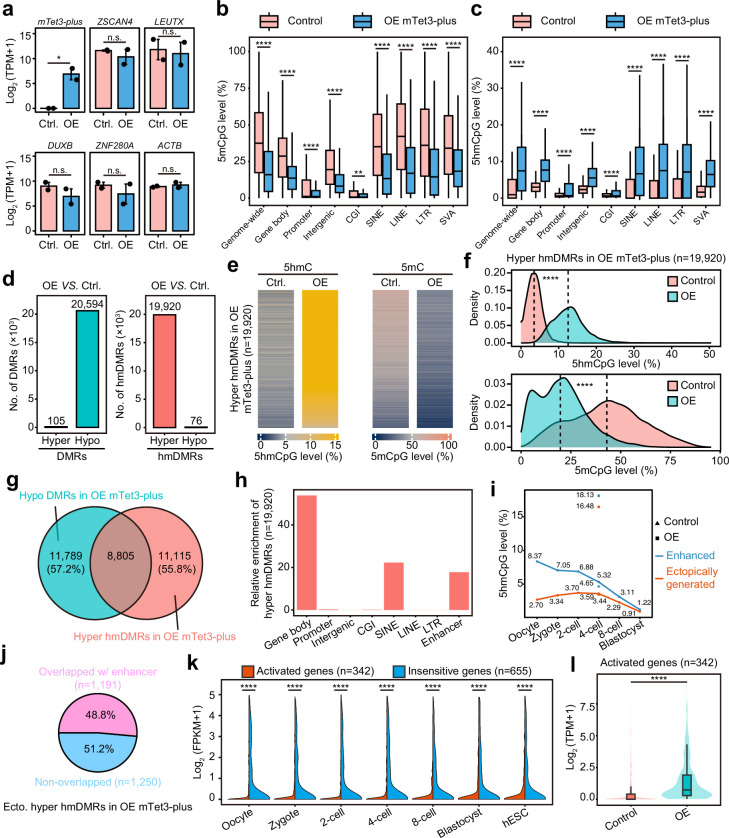
Ectopic expression of mouse enhanced Tet3 in human early embryos. **a**, Bar plots showing the expression level of mouse enhanced *Tet3* (*mTet3-plus*), human 8-cell stage marker genes (*ZSCAN4*, *LEUTX*, *DUXB* and *ZNF280A*) and housekeeping gene (*ACTB*) in control and mTet3-plus overexpressed human 8-cell embryos. There are two biological replicates in control and mTet3-plus overexpressed group, respectively. P-value were calculated by two-sided Student’s t test. Data are presented as mean values ± SEM. **b,c**, Box plots showing the 5mCpG (**b**) and 5hmCpG (**c**) levels on different genomic elements of control and mTet3-plus overexpressed human 4-cell embryos. Genome wide, genome was divided into non-overlapped 1-kb tiles. P-value were calculated by two-sided Wilcoxon signed-rank test. **d**, Bar plots showing the number of differentially DNA methylated or hydroxymethylated regions (DMRs or hmDMRs) between control and mTet3-plus overexpressed human 4-cell embryos. *VS*. denotes versus. **e**, Heatmaps showing the 5hmCpG and 5mCpG levels on hyper hmDMRs identified in mTet3-plus overexpressed human 4-cell embryos when compared to control embryos. **f**, Density plots showing the 5hmCpG and 5mCpG levels on hyper hmDMRs identified in mTet3-plus overexpressed human 4-cell embryos when compared to control embryos. Dashed lines indicate the median level. P-value were calculated by two-sided Wilcoxon signed-rank test. **g**, Overlap of hypo DMRs and hyper hmDMRs identified in mTet3-plus overexpressed human 4-cell embryos when compared to control embryos. **h**, Enrichment of hyper hmDMRs identified in mTet3-plus overexpressed human 4-cell embryos on representative genomic elements. **i**, Line plot showing the dynamics of 5hmCpG levels on hyper hmDMRs of mTet3-plus overexpressed human 4-cell embryos during early embryo development. The hyper hmDMRs were divided into two groups in Fig. 5f. **j**, Pie chart showing the percentage of ectopically generated hyper hmDMRs overlapped or not overlapped with known enhancers. Human enhancer regions were obtained from Honeybadger2. **k**, Violin plots showing the expression level of activated genes and insensitive genes in human early embryonic development. P-value were calculated by two-sided Wilcoxon signed-rank test. **l**, The expression level of activated genes in control and mTet3-plus overexpressed human 8-cell embryos. Control, n = 2; mTet3-plus, n = 2. P-value were calculated by two-sided Wilcoxon signed-rank test.

Next, we employed a gain-of-function approach using a mouse Tet3 mutant (mTet3-plus) with enhanced dioxygenase activity^33^ to validate the link between 5hmC and gene expression in human early embryos (Fig. 5a and Extended Data Fig. 7c,d). This would also enable us to confirm the correlation between 5hmC and DNA demethylation in human early embryos, as well as establish the conserved targeting property shared by mouse and human TET proteins (Fig. 3 and Extended Data Fig. 4). The successful expression of mTet3-plus was testified at both the mRNA and protein levels in human embryos (Fig. 5b and Extended Data Fig. 8a), where the expected expression of human eight-cell genes was observed (Extended Data Fig. 8a). The ectopic expression of mTet3-plus resulted in an increase in genome-wide 5hmC levels and a decrease in 5mC levels across different genomic elements (Fig. 5c and Extended Data Fig. 8b,c). Then, 20,594 hypo DMRs (median length of 6,700 bp), which represented a significant decrease in 5mC levels and increase in 5hmC levels, were identified in mTet3-plus-overexpressed human embryos (Fig. 5d,e, Extended Data Fig. 8d and Supplementary Table 6), demonstrating the firm association between 5hmC and DNA demethylation.

A total of 19,920 hyper hmDMRs (median length of 5,800 bp) were then defined in mTet3-plus overexpressed human embryos, compared with the control group (Extended Data Fig. 8d and Supplementary Table 7). These hyper hmDMRs also exhibited a significant reduction in 5mC levels (Extended Data Fig. 8e–g), and their distribution was enriched at gene bodies, SINEs and enhancers (Extended Data Fig. 8h), mirroring the pattern observed in oocyte hyper hmDMRs (Extended Data Fig. 3a). Among them, 87.7% hyper hmDMRs (*n* = 17,473) exhibited significant levels of 5hmC in natural human oocytes and zygotes (Fig. 5f and Extended Data Fig. 8i), providing evidence for conserved targeting activity between mouse and human TET protein as shown in Fig. 3. Of note, 2,441 hyper hmDMRs were ectopically generated (Fig. 5f and Extended Data Fig. 8i), with approximately half of them overlapping with known human enhancers (Extended Data Fig. 8j), thereby correlating with 1,117 nearby genes. Overall, 655 genes displayed insensitivity to elevated changes in 5hmC levels, consistently exhibiting high expression in oocytes and early human embryos (Extended Data Fig. 8k). While 342 genes that were generally low in expression or silenced in natural human embryos, became activated in mTet3-plus overexpressed embryos, including *TNS4* and *TMEM270* (Fig. 5g,h, Extended Data Fig. 8k,l and Supplementary Table 8). These results together demonstrated the close involvement of 5hmC in gene activation in early human embryos.

### 5hmC is highly enriched at OTX2 binding motifs in humans

**Fig. 6:**
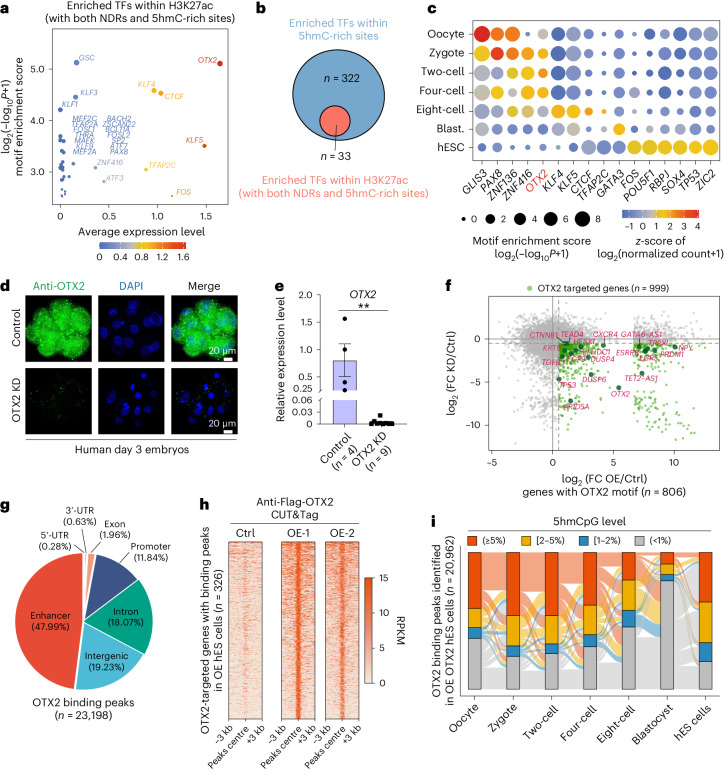
The role of DNA hydroxymethylation in transcription factor guided trans-regulation and early lineage commitment. **a**, Representative TFs in motif enrichment of eight-cell H3K27ac peaks overlapping with distal NDRs and with 5hmCpG levels (>5%). Enrichment scores (*P* value) were calculated by HOMER and were size-coded, and expression levels for TFs in human eight-cell embryos were colour-coded. **b**, Overlap of enriched TFs within 5hmC-rich sites and TFs identified in **a**. **c**, Representative TFs in motif enrichment of high-5hmC-fraction tiles. Enrichment scores were size-coded and expression levels for TFs in human eight-cell embryos were colour-coded. **d**, OTX2 staining of control (negative control siRNA microinjected) and OTX2 knockdown (KD) human day 3 embryos. Representative images from three independent biological replicates are shown. **e**, Knockdown of OTX2 in human day 4 embryos were successfully verified by RT–qPCR, compared with a negative control siRNA-injected sample. Error bars represent the mean values ± s.e.m. *P* = 0.0013, was calculated by two-sided Student’s *t*-test. **f**, Scatter-plot comparing mRNA-level changes of OTX2 KD and control human embryos on day 4 (*y* axis) and mRNA-level changes of OTX2 OE and control hES cells (*x* axis). For RNA-seq of control and OTX2 OE hES cells, *n* = 3, respectively. For RNA-seq of negative control and OTX2 knockdown human day 4 embryos, *n* = 12 and 9, respectively. **g**, Pie chart showing the proportion of OTX2 binding peaks in each genomic element. **h**, Heatmap showing OTX2 targeted genes containing its binding peaks in OE hES cells. **i**, Sankey plot showing the change of 5hmC level at OTX2 binding peaks across human early embryo development. Source data

**Extended Data Fig. 9:**
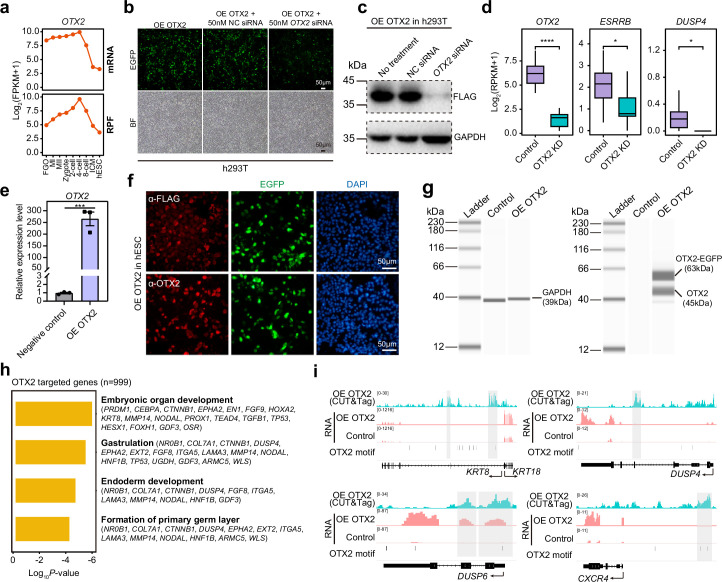
Validation of OTX2 knockdown in early human embryos and OTX2 overexpression in hESC. **a**, Transcription and translation dynamics of OTX2 in human early embryos. RPF, ribosome-protected fragments. The expression dataset for human oocytes and preimplantation embryos was retrieved from GSE197265. **b**, OTX2 overexpressed h293T cells were used to verify the knockdown efficiency of *OTX2* siRNA. Negative control siRNA and samples without siRNA treatment were used as negative controls, EGFP was co-translated with OTX2. BF, bright field. OE, overexpression. Representative images from two independent biological replicates were showed. **c**, Verification of OTX2 knockdown efficiency by Western blot. Anti-Flag antibody was used to detected flag-tagged OTX2 protein. GAPDH was used as loading control. Representative images from two independent biological replicates were showed. **d**, Representative down-regulated genes after knockdown of OTX2 in human day 4 embryos. The number of biological replicates in each sample were: control, n = 12; OTX2 KD, n = 9. Two-sided Wilcoxon signed-rank test were performed and P-value were as indicated. **e**, Significant upregulation (>250-fold) of *OTX2* in transiently transfected human ES cells was verified by RT‒qPCR. Three independent biological replicates were performed. Error bars represent the mean values ± SEM. P = 0.0008, was calculated by two-sided Student’s t-test. **f**, Verification of successful overexpression of flag-tagged OTX2 in hESCs by immunofluorescence staining. EGFP was co-translated with OTX2. **g**, EGFP-positive human ESCs were sorted by FACS and lysed for Western blot analysis. Anti-FLAG antibody was used to detect the overexpressed OTX2 (45 kDa) and OTX2-EGFP fusion protein (63 kDa). Representative images from two independent biological replicates were showed. The images are already uncropped source data. **h**, Representative Gene Ontology (GO) terms for OTX2 targeted genes. P-value were calculated by Metascape. **i**, Representative up-regulated genes in hESCs with overexpression of 3×Flag-OTX2. The OTX2 binding peaks were shaded marking. Data were obtained from CUT&Tag of OTX2-overexpressed hESCs. Source data

To further elucidate the potential *trans*-regulatory factors that cooperate with 5hmC in affecting gene expression, active enhancers with 5hmC enrichment in eight-cell human embryos (*n* = 1,186) were subjected to motif enrichment analysis to identify the corresponding transcription factors (TFs) (Fig. 6a). Six TFs yielded high enrichment scores, of which TFAP2C, KLF4/5 and CTCF were shared by both humans and mice (Fig. 6a), playing roles in early lineage differentiation^34–38^ or naive pluripotency^39^. To identify more TFs that may bind to 5hmC-enriched regulatory elements to regulate transcription in early human embryos, we performed motif enrichment analysis of genome-wide 1-kb tiles (*n* = 203,306) with significantly high levels of 5hmC in each stage (Fig. 6b,c and Extended Data Fig. 6c). Notably, several putative regulatory TFs in early development were specific to human embryos and not shared with mice, such as OTX2 (Fig. 6c). These TFs including OTX2 were lowly expressed in human ES cells (Fig. 6c and Extended Data Fig. 9a), but their targeted enhancers and motifs were still enriched with 5hmC (Fig. 6a–c).

### Knockdown of OTX2 in early human embryos

As OTX2 showed the most highly motif enrichment at 5hmC-riched enhancers (Fig. 6a), we next explored its role, which has not been documented in human early embryos.

OTX2 was translated beginning in oocytes and reached its peak level at the 4–8 cell stages (Extended Data Fig. 9a). We then used small interfering RNA (siRNA) to knockdown (KD) *OTX2* and validated its silencing efficiency in h293T cells with transient OTX2 overexpression (Extended Data Fig. 9b,c). The KD of OTX2 in human embryos was successfully achieved (Fig. 6d,e). Those downregulated genes in OTX2 KD embryos (Extended Data Fig. 9d) were further overlapped with upregulated genes in OTX2 overexpressed human ES (hES) cells (Extended Data Fig. 9e–g and Supplementary Fig. 1). This resulted identifying 999 of OTX2-targeted genes in human early embryos (Fig. 6f, Extended Data Fig. 9h and Supplementary Table 9), including *ESRRB*, *DUSP4* and *TEAD4*, which function in early embryonic development^40^. Among them, 806 genes had an OTX2 motif in potential CREs and could be regulated by it (Fig. 6f).

**Extended Data Fig. 10:**
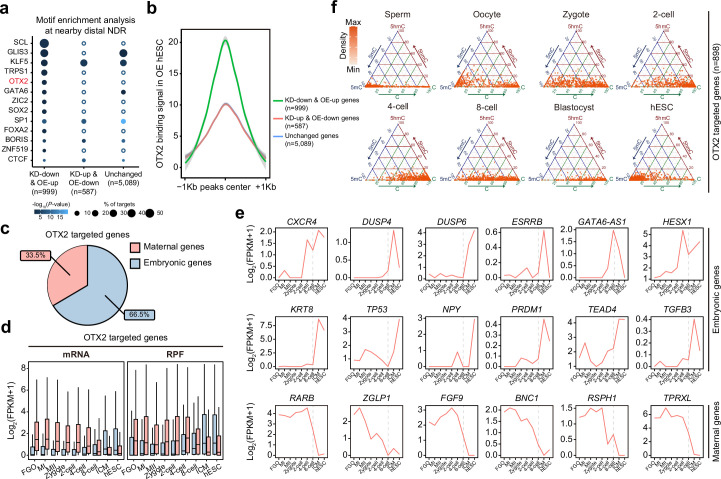
The characteristics of OTX2 targeted genes in human early embryos. **a**, Motif enrichment in the nearby distal open regions (based on scCOOL-seq data of human early embryos, GSE100272) of OTX2 targeted genes (down-regulated after OTX2 knockdown in human day 4 embryos (KD-down) and up-regulated after OTX2 overexpression in hESCs (OE-up)), KD-up & OE-down genes and unchanged genes, these genes were defined in Fig. 6f. **b**, The OTX2 signal intensity on OTX2 binding peaks linked with three types of genes after OTX2 knockdown in early human embryo and OTX2 overexpression in hESC. **c**, Pie chart shows the proportion of maternal and embryonic genes in the OTX2 targets. **d**, Expression dynamics of maternal or embryonic genes of OTX2 targets in human oocyte, preimplantation embryos and hESC. The transcriptome and translatome data for human oocytes, preimplantation embryos and hESC was retrieved from GSE197265. There are two biological replicates in each stage. RPF, ribosome-protected fragments. **e**, Expression dynamics of representative maternal or embryonic genes which were targeted by OTX2 in human oocyte, preimplantation embryos and hESC. **f**, Ternary plots showing the levels of unmodified cytosines, 5mC and 5hmC at 500 bp upstream and downstream of OTX2 binding motifs of its targeted genes in human gametes, preimplantation embryos and hESC.

To further validate that those OTX2-targeted genes could bond with OTX2 at their CREs, we performed a Cleavage Under Targets and Tagmentation assay (CUT&Tag) in OTX2 ectopically expressed hES cells (Fig. 6g,h and Extended Data Fig. 9i). We found that OTX2 was preferentially enriched at enhancers (47.99%), intergenic regions (19.23%) and introns (18.07%) (Fig. 6g). Among the OTX2-targeted genes, 326 out of 999 genes (32.6%) were bound by OTX2 in overexpressed hES cells (Fig. 6h). These genes included *KRT8*, *DUSP4*, *DUSP6* and *CXCR4*, which play functional roles in specification of trophectoderm cells^41^, differentiation of primitive endoderm cells^42^ or migration of mesoderm and definitive endoderm cells^43^ during human and mouse embryonic development (Extended Data Fig. 9i). Additionally, the OTX2 binding peaks were significantly enriched with 5hmC in both hES cells and human early embryos (Fig. 6i). Those OTX2 targeted genes (KD down and OE up) were enriched for OTX2 motifs in their linked distal NDRs in human early embryos (Extended Data Fig. 10a). And the OTX2-targeted genes of human early embryos were indeed preferentially bound by OTX2 in overexpressed hES cells (Extended Data Fig. 10b). Moreover, 66.5% of the OTX2-targeted genes were activated in the embryonic stage, whereas 33.5% were maternally expressed, suggesting an important role of OTX2 in human early development (Extended Data Fig. 10c–e).

### The link between 5hmC, OTX2 and embryonic gene expression

**Fig. 7:**
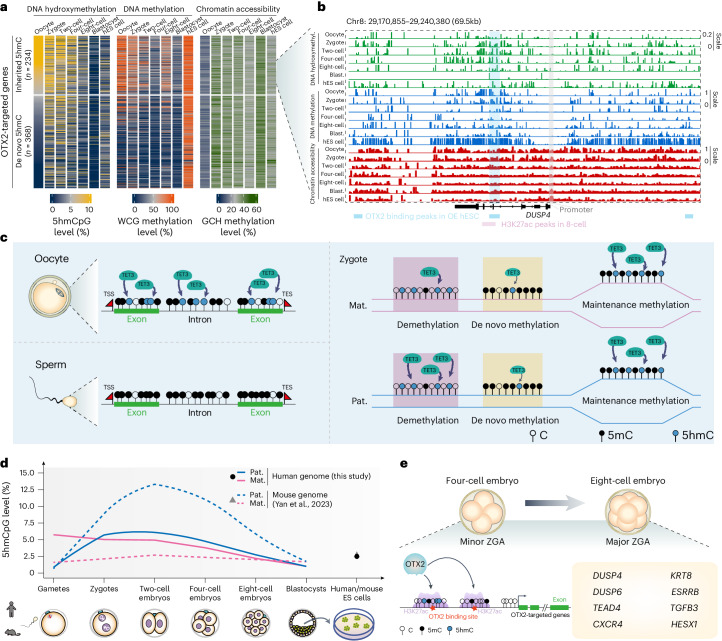
Model and mechanistic insights of the DNA hydroxymethylation dynamics during human early embryo development. **a**, Levels of DNA hydroxymethylation, DNA methylation (WCG methylation, W = A or T) and chromatin accessibility (GCH methylation, H = A, C or T) on OTX2 motif regions (500 bp upstream and downstream of motif regions) of its targets. **b**, Levels of DNA hydroxymethylation (hydroxymethyl.), DNA methylation and chromatin accessibility on OTX2-binding regions and H3K27ac-modified regions of the *DUSP4* locus in human gametes, preimplantation embryos and hES cells. **c**, Sketch of the origin, inheritance of 5hmC and its relationship with the 5mC turnover in human zygotes. Mat., maternal; Pat., paternal. **d**, Schematic of parental allele-specific 5hmCpG dynamics in human and mouse gametes, preimplantation embryos and ES cells. **e**, Model of 5hmC in association with active enhancers and linking to ZGA in human early embryos.

Last, we examined the relationship of 5hmC, OTX2-binding and chromatin status of OTX2 targeted genes (Fig. 7a), exemplified by the *DUSP4* locus (Fig. 7b). Although OTX2 targeted genes showed richness in 5hmC in corresponding enhancer regions during human early development (Extended Data Fig. 10f), they were divided into two groups based on their 5hmC level in MII oocytes (Fig. 7a). The enhancer regions of *DUSP4* became accessible and kept high levels of 5hmC after fertilization until the eight-cell stage, coinciding with the establishment of H3K27ac modifications (Fig. 7b). These results suggested that 5hmCs were primed to be generated in enhancers of lineage-specific genes that were targeted by OTX2 and linked to the regulation of corresponding gene expression in early human embryos.

## Discussion

In this study, we showed that the originations and dynamics of 5hmC during early embryos development were not conserved between humans and mice (Fig. 7c,d). Notably, human oocytes acquired remarkable levels of 5hmC during their growth (Fig. 7c,d), which may be caused by the differences in the deposition of histone modifications between human and mouse oocytes. Of the histone marks, H3K9me3 is less enriched in human oocytes than in mice and gradually occupies a greater proportion of the genome after the four-cell stage^27,28,44^. On the other hand, the establishment of the 5hmC landscape in human oocytes is firmly linked with de novo DNA methylation, as highly methylated gene bodies bear abundant 5hmC. This establishment process is independent of DNA replication, as oocytes are in meiotic arrest and resumption. Moreover, the DNA methylome of human oocytes is orchestrated by chromatin accessibility, active transcription, histone modifications such as H3K36me3 (ref. ^22^) and human-specific endogenous retroviruses^45^; thus, these factors may also influence the 5hmC programme during oocyte maturation.

In humans, OTX2 is maternally expressed from the oocyte stage^22^ and highly abundant before the blastocyst formation (Extended Data Fig. 9a). Unlike humans, mouse Otx2 could not be detected during oogenesis^22^ but shows specific expression only in post-implantation epiblasts and limits the fate of germline cells^46^. Despite the different originations of parental 5hmC in human early embryos (Fig. 7c,d), those 5hmC loci were highly enriched in the OTX2 motif. We established the link between 5hmC, OTX2, CREs and gene activation in human preimplantation embryos (Fig. 7e). Of note, mouse epiblasts were also enriched with 5hmC, which is preferentially distributed at enhancer regions^18^. The connection of 5hmC and OTX2 regulation unravelled in this work may also exist in mouse epiblasts, which is worthy of examination in a future study.

Another difference between humans and mice is that both parental genomes of early human embryos are relatively stable in genome-wide 5hmC levels until the two-cell stage. Typical DNA methylation-related factors could be detected in human early embryos at both the mRNA and protein level (Extended Data Fig. 4a,b). Additionally, *TDG*, which mediates active DNA demethylation through the BER pathway, exhibits no mRNA expression in humans or mice before the eight-cell stage (Extended Data Fig. 4a). The investigation of these factors’ role in retaining 5hmC at specific loci until the eight-cell embryo stage is crucial, as 5hmC is associated with active enhancers and linked to the activation of embryonic genes (Fig. 7e). Last, the unique distribution of 5hmC in early human embryos also make it a molecular benchmark for evaluating in vitro-derived human embryonic models in the future.

## Methods

### Experimental model and participant details

#### Ethics statement

This research was designed to study the dynamics and regulatory mechanisms of DNA hydroxymethylation in human preimplantation embryos and germ cells. The study analysed the DNA hydroxymethylation dynamics in sperm, MII oocytes, zygotes, two-cell, four-cell, eight-cell embryos, blastocysts and hES cells. Also, 3PN embryos were collected for functional verification experiments by using RNA-seq, APOBEC-coupled epigenetic sequencing (ACE-seq), whole genome bisulfite sequencing (WGBS) and immunofluorescence. The embryos were cultured in vitro for a maximum of 6 days. All the experimental procedures mentioned above have been reviewed and approved by the Biomedical Ethics Committee of Anhui Medical University (83220416). Written informed consent was obtained from all donors before enrolling in the study. Before signing informed consent, persons donating germ cells were provided with all the necessary information, including an introduction to this research, usage of donated samples, protection of privacy, a means to receive counselling, as well as the risk, gain and right of participation. Additionally, an opportunity for refusal to participate in the research was guaranteed by an opt-out. This research was conducted ethically in accordance with the measures of the People’s Republic of China on the administration of Human Assisted Reproductive Technology, the ethical principles of the Human Assisted Reproductive Technology and the Human Sperm Bank as well as the Declaration of Helsinki. Experiments on human early embryos and hES cells also followed the 2016 Standards for Human Stem Cell Use in Research issued by the International Society for Stem Cell Research.

#### Statement on exclusion of samples and data

No statistical methods were used to predetermine the sample size. In all the experiment included in this study, human early embryos, hES cells and h293T cells were collected and randomly allocated to control and treatment group without a preconceived selection strategy or prioritization by morphology or state. Data collection and analysis were not performed blind to the conditions of the experiments. No early human embryo samples or data points were excluded from the analyses for any reason.

#### Collection of human gametes and preimplantation embryos

Healthy sperm from adult donors was collected at The First Affiliated Hospital of Anhui Medical University clinic. Fresh mature oocytes and immature oocytes were voluntarily donated from women undergoing regular IVF treatment. Mature MII oocytes were randomly picked and transferred to lysis buffer for the optimized ACE-seq. The embryos were obtained through intracytoplasmic sperm injection (ICSI) using fresh or frozen–thawed sperm; this procedure was part of the research approval review and donor consent process. Fertilization was determined by noting the presence of two parental pronuclei and second polar body extrusion around 18 h after ICSI. Zygotes were collected 18–20 h after ICSI. The two-cell embryos were collected 24–28 h after ICSI. The four-cell embryos were collected on day 2 after ICSI. The eight-cell embryos were collected on day 3 after ICSI. Blastocysts were collected between day 5 and day 6 after ICSI. Before transferring into lysis buffer, zona pellucidae of zygotes, two-cell embryos, four-cell embryos, eight-cell embryos and blastocysts were removed by brief exposure to 0.5% protease (Sigma, cat. no. P8811-16). Then, the zona-free embryos were manually removed the polar bodies by a 30-μm biopsy pipette. For optimized ACE-seq, the average number of oocytes, zygotes, two-cell, four-cell, eight-cell embryos and blastocysts that were pooled as one biological replicate was 28, 24, 11, 6, 3 and 1, respectively.

### Method details

#### Construction of the ACE-seq libraries

Genomic DNA (gDNA) was extracted from human sperm and hES cells by using DNeasy Blood & Tissue kits (QIAGEN, cat. no. 69504) according to the manufacturer’s protocol, 200 ng gDNA was then subjected to the optimized ACE-seq protocol. Human oocytes and preimplantation embryos were collected as described above. The gDNA was first fragmented and then subjected to the ACE-seq library construction protocol^18^ with modifications. In brief, fragmented gDNA was glycosylated by using UDP-glucose and T4 Phage β-glucosyltransferase (New England Biolabs, cat. no. M0357) at 37 °C for 1 h. Afterwards, 0.1 M NaOH was added for denaturation at 55 °C for 10 min. The APOBEC enzyme (New England Biolabs, cat. no. E7125) was used to deaminate gDNA at 37 °C for 3 h. After deamination, gDNA was purified once using AMPure XP beads (Beckman Coulter, cat. no. A63882) before library construction following the TAILS procedure^18,22,47^. A Fragment Analyzer (Agilent 5200) was used to check the size distribution of the final libraries. Finally, the ACE-seq libraries were sequenced on a NovaSeq 6000 sequencer by a 150-bp paired-end sequencing strategy. Additionally, in vitro CpG-methylated lambda DNA (Thermo Fisher Scientific, cat. no. SD0021) was used as a spike-in to estimate the deamination rate in the 5mCpG context and unmodified CH context (H = A, C, T). In addition, we incorporated fully hydroxymethylated DNA to assess the protection rate of 5hmC by glycosylation in individual samples.

#### Construction of the WGBS libraries

Human 3PN eight-cell embryos (three embryos were pooled as one biological replicate), 200 ng of purified gDNA of hES cells or h293T cells was subjected to bisulfite conversion and purification by using the EZ-96 DNA Methylation-Direct MagPrep kit (Zymo Research, cat. no. D5045) according to the manufacturer’s protocol. Then, WGBS libraries were constructed via our recently published TAILS method^22,47^. In brief, P5-N6-oligo1 (5′-CTACACGACGCTCTTCCGATCTN_6_-3′) was used for the first round of random priming in the presence of the Klenow exo(-) fragment (QIAGEN, cat. no. P7010-HC-L). The remaining oligonucleotides and dNTPs were removed by Exo-SAP IT Express (Applied Biosystems, cat. no. 75001) and the dC tailing step was performed with the TdT enzyme (Thermo Fisher, cat. no. EP0162). Then, a second round of priming was conducted by using P7-G6-oligo2 (5′-AGACGTGTGCTCTTCCGATCTG_6_HN-3′) in the presence of the Klenow exo(-) fragment. After one round of purification with AMPure XP beads, libraries were constructed by PCR amplification. Next, the libraries were purified and the size distribution was checked on the Fragment Analyzer. Finally, the WGBS libraries were sequenced on a NovaSeq 6000 sequencer with a 150-bp paired-end sequencing strategy.

#### Construction of RNA-seq libraries

Nearly 100 enhanced green fluorescent protein (eGFP)-positive hES cells or single human embryos were collected as one biological replicate for RNA-seq library construction by using the Smart-seq2 method. In brief, cells or embryos were picked and transferred to lysis buffer and poly(A)^+^ RNA was captured by oligo(dT) primers. First-strand cDNA was synthesized by reverse transcription and then amplified, purified and fragmented. Fragmented cDNA was processed to construct an RNA-seq library using the NEBNext UltraII DNA Library Prep kit (New England Biolabs, cat. no. E7645L) according to the manufacturer’s protocol for sequencing on an Illumina NovaSeq 6000 sequencer with a 150-bp paired-end sequencing strategy.

#### Small molecule inhibitor treatment assay

For small molecule inhibitor treatment experiments in h293T cells, the culture medium of newly passaged h293T cells were supplemented with 150 μM DMOG (Selleck, cat. no. S7483) or 80 μM BC339 (Selleck, cat. no. S6682). Then, 0.3% DMSO (Sigma, cat. no. D2438) was used as a negative control. Cells were cultured in medium containing DMSO or small molecule inhibitors for another 2 days. The treatment effect of the inhibitors was evaluated by immunofluorescence assay of 5hmC and WGBS. For human early embryos, inhibitor treatment experiments were performed using 3PN zygotes, which were clinically discarded and donated by patients from IVF treatments after receiving signed informed consent. The 3PN zygotes were identified and collected on day 1 after IVF, then 3PN zygotes were transferred to G1plus medium (Vitrolife, cat. no. 10128) in the presence of 0.3% DMSO, 150 μM DMOG or 80 μM BC39, respectively. Embryos were cultured to day 3 for immunostaining of 5hmC or day 4 for Smart-seq2 library preparation and each single embryo was used as a biological replicate. To further verify the inhibitory activity of BC339 on TET protein, a mTet3-plus plasmid was transiently transfected into h293T cells and cultured in 80 μM BC339-containing medium. After 24 h, cells were collected for immunofluorescence staining of 5hmC and western blotting.

#### In vitro transcription and microinjection

The coding sequence of mouse enhanced *Tet3* (mTet3-plus) was synthesized and cloned into a pcDNA3.1(+) vector with hemagglutinin (HA)-tag. The mTet3-plus plasmid was linearized and mRNA was synthesized using the EasyCap T7 Co-transcription kit with CAG Trimer (Vazyme, cat. no. DD4203) according to the manufacturer’s instructions. Next, mRNA was purified by VAHTS RNA Clean Beads (Vazyme, cat. no. N412-01) and eluted by nuclease-free water. The integrity of the synthesized mRNA was confirmed by a 5200 Fragment Analyzer System (Agilent). For mRNA microinjection, human 3PN embryos were injected with approximately 10 pl mouse enhanced *Tet3* mRNA (1.5 μg μl^−1^) using a FemotoJet microinjector (Eppendorf) with constant flow setting. Then, control and injected embryos were cultured until day 2 for immunostaining of HA-mTet3-Plus, 5hmC and 5mC. Four-cell stage embryos were collected for ACE-seq and WGBS. Eight-cell stage embryos were collected for a single-embryo Smart-seq2 assay.

#### OTX2 knockdown assay

The silencing efficiency of *OTX2* siRNA (Ribobio, cat. no. SIGS0007344-4) was first tested in h293T cells with ectopic expression of Flag-tagged OTX2. In brief, Flag-tagged *OTX2* plasmids and 50 nM *OTX2* siRNA were transiently co-transfected into human 293T cells using Lipofectamine 3000 Transfection Reagent (Thermo Fisher, cat. no. L3000001); 50 nM negative control siRNA was also co-transfected with *OTX2* plasmids into h293T cells. After transfection for 24 h, the eGFP signal was observed under a fluorescence microscope and cells were collected for western blot. For OTX2 knockdown in early human embryos, *OTX2* siRNA at 50 nM was microinjected into the cytoplasm of human 3PN zygotes using an Eppendorf FemtoJet 4i. After injection, embryos were cultured in G1plus medium for subsequent experiments. The control group was microinjected with 50 nM negative control siRNA. A RT–qPCR primer was used to evaluate the knockdown or overexpression efficiency of *OTX2* (forward primer: 5′-catgcagaggtcctatcccat-3′ and reverse primer: 5′-aagctggggactgattgagat-3′).

#### Overexpression of OTX2 in hES cells

The overexpression constructs were made using a ClonExpress II One Step Cloning kit (Vazyme, cat. no. C112). The coding sequence of human *OTX2* was cloned into a pPBbsr2 vector with Flag-tag and eGFP. After translation, a 3 × Flag tag was fused with the target protein. In addition, eGFP was co-translated with the target protein and then separated by P2A self-cleaving peptide. The successfully constructed plasmid was verified by Sanger sequencing before use. To overexpress OTX2 in hES cells, plasmids were transiently transfected into hES cells using Lipofectamine Stem Transfection Reagent (Thermo Fisher, cat. no. STEM00003). After transfection for 24 h, the eGFP signal was observed in overexpressed hES cells under a fluorescence microscope. Then, the cells were digested with Versene Solution and prepared for FACS. A BD FACS Fusion instrument and BD FACSDiva 9.0 software were used to sort eGFP-positive cells. Sorted cells were kept in DPBS supplemented with 0.04% BSA and ROCK inhibitor (10 μM Y-27632, Selleck, cat. no. s1049) for western blot, RNA-seq and CUT&Tag analysis.

#### CUT&Tag of OTX2-overexpressed hES cells

A Hyperactive Universal CUT&Tag Assay kit (Vazyme, cat. no. TD904) was used to construct the CUT&Tag libraries of OTX2-overexpressed hES cells according to the manufacturer’s protocol. The hES cells that were not transfected with OTX2-overexpression plasmid were used as a negative control. In brief, 100,000 eGFP-positive OTX2-overexpressed hES cells or control hES cells were washed and resuspended in 100 μl wash buffer before 10 µl activated concanavalin A-coated magnetic beads were added to each sample and incubated at room temperature for 10 min. After that, cells were incubated with 4 µg anti-Flag antibody (Sigma, cat. no. F7425) on a rotating platform for 2 h at room temperature. Then, anti-rabbit IgG antibody (Abcam, cat. no. ab6702, 1:100) was added and incubated at room temperature for 60 min. Next, cells were washed three times with Dig-Wash buffer to remove unbound antibodies. After that, pA/G-Tnp incubation was performed on a rotating platform for 1 h at room temperature. Similarly, cells were washed three times with Dig-300 buffer to remove unbound pA/G-Tnp protein and resuspended in tagmentation buffer and incubated at 37 °C for 1 h. Tagmentation was stopped by adding of 10% SDS and incubating at 55 °C for 10 min. Then cells were plated on a magnet rack, supernatant was transferred into a new PCR tube and DNA was extracted by using DNA Extract Beads Pro. Finally, libraries were constructed using TruePrep Index Kit V2 for Illumina (Vazyme, cat. no. TD202). All CUT&Tag libraries were sequenced using the Illumina NovaSeq 6000 platform with a PE150 sequencing strategy.

#### Immunofluorescence staining assay

For immunostaining of cultured hES cells or h293T cells, sterile glass coverslips were plated into culture dishes. Cells were seeded into dishes and cultured to the appropriate density at 37 °C. After that, cells were collected and fixed. For immunostaining of human zygotes, day 2 and day 3 embryos, zona pellucidae were first removed with 0.5% pronase (Sigma, cat. no. P8811). Embryos were fixed with 4% freshly prepared paraformaldehyde (Sigma, cat. no. P6148) at room temperature for 30 min. After fixation, samples were washed three times with wash buffer (DPBS with 0.05% Tween-20) for 5 min each time. Then, samples were permeabilized with 0.2% Triton X-100 at room temperature for 30 min. For detection of 5hmC and 5mC, samples were treated with 4 N HCl at room temperature for 15 min and subsequently neutralized with 100 mM Tris-HCl buffer (pH 8.0) for 10 min. Samples were blocked in block solution (DPBS with 2% BSA, 0.05% Tween-20 and 1% goat serum) at room temperature for 1 h and incubated with primary antibody at 4 °C overnight. After washing, samples were further incubated with secondary antibody at room temperature for 1 h. Finally, samples were washed and mounted by using ProLong Gold Antifade Mountant with DAPI (Thermo Fisher, cat. no. P36931). All immunofluorescence images were taken using a Zeiss LSM-880 confocal microscope with ZEN (2012 SP2) for data collection. The primary antibodies used in this study were anti-OTX2 (Santa Cruz, cat. no. sc-514195, 1:200 dilution); anti-Flag (Sigma, cat. no. F7425, 1:500 dilution); anti-HA (Abcam, cat. no. ab18181, 1:500 dilution); anti-5hmC (Active Motif, cat. no. 39791, 1:500 dilution); and anti-5mC (Diagenode, cat. no. C15200081-100, 1:500 dilution). Secondary antibodies used were goat anti-mouse Alexa Fluor 488 (Invitrogen, cat. no. A11029, 1:1,000 dilution) and goat anti-rabbit Alexa Fluor 555 (Invitrogen, cat. no. A21429, 1:1,000 dilution).

#### Immunoblotting assay

The eGFP-positive hES cells were lysed in RIPA lysis buffer (Beyotime, cat. no. P0013B) and analysed using ProteinSimple Wes Western Blot System (data collection software, Compass for SW v.4.1.0). Transfected h293T cells were collected and then lysed in RIPA lysis buffer containing 1 mM PMSF (Thermo Fisher Scientific, cat. no. 36978). Then 5× loading buffer (Solarbio, cat. no. P1040) was added to a final concentration of 1× and samples were denatured at 95 °C for 10 min. Lysate samples were loaded on a 8% or 10% SDS–PAGE gels and electrophoretically transferred to nitrocellulose membrane. Next, the membrane was probed with primary antibodies against Flag (Sigma, cat. no. F1804, 1:2,000 dilution), GAPDH (Cell Signalling Technology, cat. no. 2118s, 1:1,000 dilution), HA (Abclonal, cat. no. AE008, 1:2,000 dilution), HRP-conjugated β-actin (Proteintech, cat. no. HRP-60008, 1:2,000 dilution) at 4 °C overnight and incubated with secondary antibody against rabbit IgG (horseradish peroxidase-conjugated; Proteintech, cat. no. SA00001-2, 1:1,000 dilution), mouse IgG (horseradish peroxidase-conjugated; Proteintech, cat. no. SA00001-1, 1:1,000 dilution) at room temperature for 1.5 h. The chemiluminescence signal was detected using 810 western ECL peroxide buffer (Tanon, cat. no. 180-501W) and luminol/enhancer solution (Tanon, cat. no. 180-501B).

### Quantification and statistical analysis

#### Processing ACE-seq and WGBS data

Raw 150-bp paired-end reads were trimmed using TrimGalore (v.0.6.6) with the following parameters: –quality 20 –stringency 3 –length 50 –clip_R1 9 –clip_R2 9 –paired –trim1 –phred33. Adaptors and low-quality reads were filtered out. The remaining reads were aligned to the reference genome (UCSC hg19) using Bismark (v.0.23.1). The alignment process was carried out in paired-end mode initially, followed by two independent rounds of alignment in single-end mode for unmapped Read1 and Read2. The deduplication of the resulting reads was performed using sambamba markup (v.0.8.2), and the function filter_non_conversion in Bismark was utilized to filter out reads with three consecutive non-conversion sites. The remaining reads were then subjected to downstream analysis. MethylDackel (v.0.5.0) was used to extract and count cytosines in the CpG sequence contexts (where H = A, T or C). The 5mC/5hmC level was defined as the percentage of C/(C + T) at each site.

#### Identifying differentially methylated regions

Replicates were aggregated to identify differentially methylated regions (DMRs) as previously described. The human genome was divided into windows of 100 bp. Only windows covering at least two CpG sites were retained. A Student’s *t-*test for differences was carried out on sliding windows of 1,000 bp length and 300 bp step size. Overlapping tiles with *P* values <0.1 were merged. To identify DMRs, tiles were compared between two groups (level in A minus level in B). If the differences in the average CpG methylation level on a specific tile were more than 0.25, this tile was labelled hyper DMR. When the differences were less than −0.25, this tile was labelled hypo DMR. The cutoffs for identifying hmDMRs were 0.1 and −0.1, respectively. Regions within 10 kb that were labelled as the same type were merged. Regions that covered at least three CpG sites in the aggregated data and had significant differences (false discovery rate-adjusted multiple *t*-test) were retained for further analysis. The adjusted *P* value cutoff was <0.01 for DMRs and <0.025 for hmDMRs.

#### Subtracting 5hmC from WGBS data by combining ACE-seq data

For an individual CG site, the 5hmC level was subtracted from the WGBS data using the MLML tool (v.5.0.0). The methylation level determined by WGBS and the level of 5hmC determined by ACE-seq were the inputs for the MLML tool (v.5.0.0). The levels of 5mC and unmodified CG were estimated. CG sites with a negative level or any conflict were excluded.

#### Definition of high-confidence 5hmCpG sites

High-confidence 5hmCpG sites were defined as previously described with minor adjustments. For individual CpG sites, the number of C and T bases from ACE-seq reads were counted. The C in a read represented 5hmC and the T represented methylated or unmodified cytosines. The averaged non-conversion rate for 5mCG was estimated using the CpG level in the λDNA spike-in and was considered the error rate of A3A deamination. Thus, high-confidence 5hmCpG sites were called based on the binominal distribution as previously reported. CpG sites with *P* values <0.025 were considered as high-confidence 5hmCpG sites.

#### Processing CUT&Tag data

Adaptors were trimmed from raw sequences using Trim Galore (v.0.6.6). The trimmed reads were aligned to the human reference genome (hg19) using bowtie2 with parameters: –local –very-sensitive –no-mixed –no-discordant –phred33 -I 10 -X 700. PCR duplicates were identified and removed using sambamba (v.0.8.2). Unmapped reads were further excluded using samtools (v.1.17). Read pairs that were on the same chromosome and fragment length less than 1,000 bp were used for the downstream analysis. Peak calling was performed using SEACR (v.1.3). The enriched regions in OTX2-overexpressed hES cells were called using the wild-type hES cells as a control track.

#### Quantifying the expression in RNA-seq data

Raw reads were retrieved from published datasets. Alignment was performed using STAR (v.2.7.10b). The UCSC hg19 and mm9 genome sequences were used for *Homo* *sapiens* and *Mus* *musculus*, respectively. Raw counts for gene expression were quantified using featureCounts (v.2.0.4). Annotations were retrieved from UCSC genome browsers and TEtranscripts. Gene Ontology enrichment analysis was performed with Metascape.

#### Definition of high-5hmC-fraction tiles

The human genome was divided into windows of 1 kb. The average 5hmC level on an individual tile was calculated, and the number of significant 5hmCpGs was counted. Tiles with coverage of at least three significant 5hmCpG sites and a 5hmC level greater than 5% were defined as high-5hmC-fraction tiles.

#### Motif enrichment analysis

Motif enrichment analysis was further performed using findMotifisGenome.pl in HOMER (v.4.11). Parameters for high-5hmC-fraction tiles were -size 2000 -len 8 -S 100 and the default for distal NDRs.

#### Inferring transcription factors binding sites

The motif matching tool MOODS (v.1.9.4) was used to inferring genome-wide TF binding sites (*P* value < 10^−5^). The raw position frequency matrix for OTX2 was retrieved from JASPAR with accession code MA0712.2. Genes with putative TFs binding sites within TSS upstream 15 kb were considered as target genes of TFs.

#### Enrichment of 5hmCpG sites and DMRs at genomic elements

The enrichment of high-confidence 5hmCpG sites within different genomic elements was quantified as follows: the number of covered high-confidence 5hmCpG sites was counted and normalized by the number of detected 2× 5hmCpG sites (in megabases) and the length of the element (in megabases). To quantify the enrichment of DMRs at genomic elements, Fisher’s exact test was performed on the length of DMRs and the genome, and the −log_10_(*P* value) was defined as the enrichment score.

#### Statistical analysis

For boxplots, the lower and upper boundaries of the boxes represent the 25th and 75th percentiles, respectively and the line inside the box represents the median value. The ends of the whiskers indicate the highest data values within the 1.5-fold interquartile range of the 75th percentile value and the lowest data values within the 1.5-fold interquartile range of the 25th percentile value. For bar plots, error bars represent the mean ± s.e.m. Data distribution was assumed to be normal but this was not formally tested. *P* values were calculated by a two-sided Wilcoxon’s test or Student’s *t*-test as indicated. *P < 0.05; **P < 0.01; ***P < 0.001; ****P < 0.0001; NS, not significant. For genomic track plots, values were scaled to the range 0–1 or 0–0.2, as indicated.

### Resource availability

#### Materials availability

This study did not generate new unique reagents.

### Reporting summary

Further information on research design is available in the Nature Portfolio Reporting Summary linked to this article.

## Online content

Any methods, additional references, Nature Portfolio reporting summaries, source data, extended data, supplementary information, acknowledgements, peer review information; details of author contributions and competing interests; and statements of data and code availability are available at 10.1038/s41556-024-01475-y.

## Supplementary information

Supplementary Information Supplementary Methods, Supplementary Figure, Statistics Source Data Table and legends for Supplementary Tables.

Reporting Summary

Supplementary Tables Supplementary tables and associated legends.

## Source data

Source Data Extended Data Fig. 7 Unprocessed western blots of Extended Data Fig. 7.

Source Data Extended Data Fig. 9 Unprocessed western blots of Extended Data Fig. 9.

Source Data Fig. 6 and Extended Data Figs. 4, 5 and 9 qPCR source data and bar plots source data of Fig. 6 and Extended Data Figs. 4, 5 and 9.

## Data Availability

Sequencing data that support the findings of this study have been deposited in the Gene Expression Omnibus under accession code GSE224618 and GSA (HRA006264) and OMIX (OMIX005397 and OMIX005398) in the National Genomics Data Center (https://bigd.big.ac.cn/). The Human reference genome hg19 was obtained from UCSC (https://hgdownload.cse.ucsc.edu/goldenpath/hg19/chromosomes/). Previously published datasets that were re-analysed here are available under the following accession codes: ACE-seq data of mouse gametes and preimplantation embryos (GSE186357)^18^, scRNA-seq data of human oocytes, preimplantation embryos and hES cells (GSE36552)^48^, Ribo-RNA-lite data of human mature oocytes, preimplantation embryos and hES cells (GSE197265)^49^, scBS-seq data of human gametes and preimplantation embryos (GSE81233)^50^, scCOOL-seq data of human gametes, preimplantation embryos and ES cells (GSE100272)^51^ and scChaRM-seq data of human growing oocytes and mature oocytes (GSE154762)^22^. Human ES cells, oocytes and early embryonic datasets with peaks of H3K4me3, H3K27me3, H3K27ac and H3K9me3 are deposited as GSE124718, GSE176016 and GSE52824 (refs. ^26,27,52^). Proteome data of human preimplantation embryos is available via the integrated proteome resources (iProX) of ProteomeXchange (PXD024267)^53^. All other data supporting the findings of this study are available from the corresponding author on reasonable request. Source data are provided with this paper.

## References

[1] Greenberg, M. V. C. & Bourc’his, D. The diverse roles of DNA methylation in mammalian development and disease. *Nat. Rev. Mol. Cell Biol.* **20**, 590–607 (2019). 31399642 10.1038/s41580-019-0159-6

[2] Suzuki, M. M. & Bird, A. DNA methylation landscapes: provocative insights from epigenomics. *Nat. Rev. Genet*. **9**, 465–476 (2008). 18463664 10.1038/nrg2341

[3] Jones, P. A. Functions of DNA methylation: islands, start sites, gene bodies and beyond. *Nat. Rev. Genet*. **13**, 484–492 (2012). 22641018 10.1038/nrg3230

[4] Rasmussen, K. D. & Helin, K. Role of TET enzymes in DNA methylation, development, and cancer. *Genes Dev.* **30**, 733–750 (2016). 27036965 10.1101/gad.276568.115 PMC4826392

[5] Schubeler, D. Function and information content of DNA methylation. *Nature* **517**, 321–326 (2015). 25592537 10.1038/nature14192

[6] Lu, X., Zhao, B. S. & He, C. TET family proteins: oxidation activity, interacting molecules, and functions in diseases. *Chem. Rev.* **115**, 2225–2239 (2015). 25675246 10.1021/cr500470n PMC4784441

[7] Parry, A., Rulands, S. & Reik, W. Active turnover of DNA methylation during cell fate decisions. *Nat. Rev. Genet*. **22**, 59–66 (2021). 33024290 10.1038/s41576-020-00287-8

[8] He, Y. F. et al. Tet-mediated formation of 5-carboxylcytosine and its excision by TDG in mammalian DNA. *Science* **333**, 1303–1307 (2011). 21817016 10.1126/science.1210944 PMC3462231

[9] Wang, D. et al. Active DNA demethylation promotes cell fate specification and the DNA damage response. *Science* **378**, 983–989 (2022). 36454826 10.1126/science.add9838 PMC10196940

[10] Wu, X. & Zhang, Y. TET-mediated active DNA demethylation: mechanism, function and beyond. *Nat. Rev. Genet*. **18**, 517–534 (2017). 28555658 10.1038/nrg.2017.33

[11] Mellen, M., Ayata, P., Dewell, S., Kriaucionis, S. & Heintz, N. MeCP2 binds to 5hmC enriched within active genes and accessible chromatin in the nervous system. *Cell* **151**, 1417–1430 (2012). 23260135 10.1016/j.cell.2012.11.022 PMC3653293

[12] Spruijt, C. G. et al. Dynamic readers for 5-(hydroxy)methylcytosine and its oxidized derivatives. *Cell* **152**, 1146–1159 (2013). 23434322 10.1016/j.cell.2013.02.004

[13] Du, Z., Zhang, K. & Xie, W. Epigenetic reprogramming in early animal development. *Cold Spring Harb. Perspect. Biol.* **14**, a039677 (2022). 34400552 10.1101/cshperspect.a039677 PMC9248830

[14] Wen, L. & Tang, F. Human germline cell development: from the perspective of single-cell sequencing. *Mol. Cell* **76**, 320–328 (2019). 31563431 10.1016/j.molcel.2019.08.025

[15] Smith, Z. D. et al. DNA methylation dynamics of the human preimplantation embryo. *Nature* **511**, 611–615 (2014). 25079558 10.1038/nature13581 PMC4178976

[16] Smith, Z. D. et al. A unique regulatory phase of DNA methylation in the early mammalian embryo. *Nature* **484**, 339–344 (2012). 22456710 10.1038/nature10960 PMC3331945

[17] Guo, F. et al. Active and passive demethylation of male and female pronuclear DNA in the mammalian zygote. *Cell Stem Cell* **15**, 447–459 (2014). 25220291 10.1016/j.stem.2014.08.003

[18] Yan, R. et al. Dynamics of DNA hydroxymethylation and methylation during mouse embryonic and germline development. *Nat. Genet.* **55**, 130–143 (2023). 36539615 10.1038/s41588-022-01258-x

[19] Shen, L. et al. Tet3 and DNA replication mediate demethylation of both the maternal and paternal genomes in mouse zygotes. *Cell Stem Cell* **15**, 459–471 (2014). 25280220 10.1016/j.stem.2014.09.002 PMC4201500

[20] Gu, T. P. et al. The role of Tet3 DNA dioxygenase in epigenetic reprogramming by oocytes. *Nature* **477**, 606–610 (2011). 21892189 10.1038/nature10443

[21] Vaisvila, R. et al. Enzymatic methyl sequencing detects DNA methylation at single-base resolution from picograms of DNA. *Genome Res*. **31**, 1280–1289 (2021). 34140313 10.1101/gr.266551.120 PMC8256858

[22] Yan, R. et al. Decoding dynamic epigenetic landscapes in human oocytes using single-cell multi-omics sequencing. *Cell Stem Cell* **28**, 1641–1656 e1647 (2021). 33957080 10.1016/j.stem.2021.04.012

[23] Guo, H. et al. The DNA methylation landscape of human early embryos. *Nature* **511**, 606–610 (2014). 25079557 10.1038/nature13544

[24] Petrussa, L., Van de Velde, H. & De Rycke, M. Similar kinetics for 5-methylcytosine and 5-hydroxymethylcytosine during human preimplantation development in vitro. *Mol. Reprod. Dev.* **83**, 594–605 (2016). 27163211 10.1002/mrd.22656

[25] Wu, J. et al. Chromatin analysis in human early development reveals epigenetic transition during ZGA. *Nature* **557**, 256–260 (2018). 29720659 10.1038/s41586-018-0080-8

[26] Xia, W. et al. Resetting histone modifications during human parental-to-zygotic transition. *Science* **365**, 353–360 (2019). 31273069 10.1126/science.aaw5118

[27] Yu, H. et al. Dynamic reprogramming of H3K9me3 at hominoid-specific retrotransposons during human preimplantation development. *Cell Stem Cell* **29**, 1031–1050 e1012 (2022). 35803225 10.1016/j.stem.2022.06.006

[28] Xu, R. et al. Stage-specific H3K9me3 occupancy ensures retrotransposon silencing in human preimplantation embryos. *Cell Stem Cell* **29**, 1051–1066 e1058 (2022). 35803226 10.1016/j.stem.2022.06.001

[29] Lv, H. et al. A small-molecule degrader of TET3 as treatment for anorexia nervosa in an animal model. *Proc. Natl Acad. Sci. USA* **120**, e2300015120 (2023). 37036983 10.1073/pnas.2300015120 PMC10120042

[30] Kusi, M. et al. 2-Hydroxyglutarate destabilizes chromatin regulatory landscape and lineage fidelity to promote cellular heterogeneity. *Cell Rep.* **38**, 110220 (2022). 35021081 10.1016/j.celrep.2021.110220 PMC8811753

[31] Beaudoin, J. D. et al. Analyses of mRNA structure dynamics identify embryonic gene regulatory programs. *Nat. Struct. Mol. Biol.* **25**, 677–686 (2018). 30061596 10.1038/s41594-018-0091-z PMC6690192

[32] Mazid, M. A. et al. Rolling back human pluripotent stem cells to an eight-cell embryo-like stage. *Nature* **605**, 315–324 (2022). 35314832 10.1038/s41586-022-04625-0

[33] Zhang, X. J. et al. Auto-suppression of Tet dioxygenases protects the mouse oocyte genome from oxidative demethylation. *Nat. Struct. Mol. Biol.* **31**, 42–53 (2024). 38177668 10.1038/s41594-023-01125-1

[34] Zhu, M. et al. Developmental clock and mechanism of de novo polarization of the mouse embryo. *Science* **370**, eabd2703 (2020). 33303584 10.1126/science.abd2703 PMC8210885

[35] Lin, S. C., Wani, M. A., Whitsett, J. A. & Wells, J. M. Klf5 regulates lineage formation in the preimplantation mouse embryo. *Development* **137**, 3953–3963 (2010). 20980403 10.1242/dev.054775 PMC2976279

[36] Jung, Y. H. et al. Maintenance of CTCF- and transcription factor-mediated interactions from the gametes to the early mouse embryo. *Mol. Cell* **75**, 154–171 e155 (2019). 31056445 10.1016/j.molcel.2019.04.014 PMC6625867

[37] Ema, M. et al. Kruppel-like factor 5 is essential for blastocyst development and the normal self-renewal of mouse ESCs. *Cell Stem Cell* **3**, 555–567 (2008). 18983969 10.1016/j.stem.2008.09.003

[38] Guo, G. et al. Klf4 reverts developmentally programmed restriction of ground state pluripotency. *Development* **136**, 1063–1069 (2009). 19224983 10.1242/dev.030957 PMC2685927

[39] Pastor, W. A. et al. TFAP2C regulates transcription in human naïve pluripotency by opening enhancers. *Nat. Cell Biol.* **20**, 553–564 (2018). 29695788 10.1038/s41556-018-0089-0 PMC5926822

[40] Gassler, J. et al. Zygotic genome activation by the totipotency pioneer factor Nr5a2. *Science* **378**, 1305–1315 (2022). 36423263 10.1126/science.abn7478

[41] Lim, H. Y. G. et al. Keratins are asymmetrically inherited fate determinants in the mammalian embryo. *Nature* **585**, 404–409 (2020). 32848249 10.1038/s41586-020-2647-4

[42] Azami, T. et al. Regulation of the ERK signalling pathway in the developing mouse blastocyst. *Development* **146**, dev177139 (2019). 31320324 10.1242/dev.177139

[43] Morrison, G. M. et al. Anterior definitive endoderm from ESCs reveals a role for FGF signaling. *Cell Stem Cell* **3**, 402–415 (2008). 18940732 10.1016/j.stem.2008.07.021

[44] Wang, C. et al. Reprogramming of H3K9me3-dependent heterochromatin during mammalian embryo development. *Nat. Cell Biol.* **20**, 620–631 (2018). 29686265 10.1038/s41556-018-0093-4

[45] Bogutz, A. B. et al. Evolution of imprinting via lineage-specific insertion of retroviral promoters. *Nat. Commun.* **10**, 5674 (2019). 31831741 10.1038/s41467-019-13662-9 PMC6908575

[46] Zhang, J. et al. OTX2 restricts entry to the mouse germline. *Nature* **562**, 595–599 (2018). 30283136 10.1038/s41586-018-0581-5 PMC6485399

[47] Gu, C., Liu, S., Wu, Q., Zhang, L. & Guo, F. Integrative single-cell analysis of transcriptome, DNA methylome and chromatin accessibility in mouse oocytes. *Cell Res.* **29**, 110–123 (2019). 30560925 10.1038/s41422-018-0125-4 PMC6355938

[48] Yan, L. et al. Single-cell RNA-seq profiling of human preimplantation embryos and embryonic stem cells. *Nat. Struct. Mol. Biol.* **20**, 1131–1139 (2013). 23934149 10.1038/nsmb.2660

[49] Zou, Z. et al. Translatome and transcriptome co-profiling reveals a role of TPRXs in human zygotic genome activation. *Science* **378**, abo7923 (2022). 36074823 10.1126/science.abo7923

[50] Zhu, P. et al. Single-cell DNA methylome sequencing of human preimplantation embryos. *Nat. Genet*. **50**, 12–19 (2018). 29255258 10.1038/s41588-017-0007-6

[51] Li, L. et al. Single-cell multi-omics sequencing of human early embryos. *Nat. Cell Biol.* **20**, 847–858 (2018). 29915357 10.1038/s41556-018-0123-2

[52] Gafni, O. et al. Derivation of novel human ground state naive pluripotent stem cells. *Nature* **504**, 282–286 (2013). 24172903 10.1038/nature12745

[53] Dang, Y. et al. Functional profiling of stage-specific proteome and translational transition across human preimplantation embryo development at a single-cell resolution. *Cell Discov.* **9**, 10 (2023). 36693841 10.1038/s41421-022-00491-2 PMC9873803

