# Supplementary material for: Distinct dynamics of parental 5-hydroxymethylcytosine during human preimplantation development regulate early lineage gene expression
Source: Nat Cell Biol. 2024 Jul 30;26(9):1458–69. doi: 10.1038/s41556-024-01475-y (PMC11392820; doi:10.1038/s41556-024-01475-y)
Supplement: Supplementary file 1 — Supplementary Methods, Supplementary Figure, Statistics Source Data Table and legends for Supplementary Tables. [file 41556_2024_1475_MOESM1_ESM.pdf]

nature cell biology

Supplementary information https://doi.org/10.1038/s41556-024-01475-y

Distinct dynamics of parental 5-hydroxymethylcytosine during human preimplantation development regulate early lineage gene expression

In the format provided by the authors and unedited

SUPPLEMENTARY METHODS

EXPERIMENTAL MODEL AND SUBJECT DETAILS

Ethics statement

Prior to commencing this project, an application must be submitted to the Biomedical Ethics Committee of Anhui Medical University. The submission should include the ethics review application form, detailed research proposal, and informed consent form. The Committee will assign at least five members from internal and external sources to review the application. Upon completion of the review, the Biomedical Ethics Committee will allow this research with significant oversight and ongoing dialogue. The First Affiliated Hospital of Anhui Medical University was responsible for recruiting oocyte and sperm donors for the study. Thirty-two oocyte donors, aged 21-40 years, were recruited from females undergoing regular IVF treatments. These patients generously donated excess eggs to support our scientific research, with no additional hormonal stimulation or egg retrieval performed solely for donation purposes. Two healthy sperm donors, aged 23 and 36 years respectively, were recruited locally through print advertisements. Study participants who provided sperm and oocytes specifically for this study received financial compensation for their time, effort, and any discomfort associated with the donation process. Females undergoing IVF treatment who donated immature oocytes and discarded 3PN embryos did not receive any financial compensation.

Cell culture

The primed human ES cell line (WIBR3) was a gift from Professor Hao-Yi Wang (State Key Laboratory of Stem Cell and Reproductive Biology, Institute of Zoology, Chinese Academy of Sciences, Beijing 100101, China). Human ES cells were cultured in Essential-8^{TM} medium (Thermo Fisher, Cat# A1517001) containing Matrigel^{TM} (Corning, Cat# 354277). Cells were passaged using Versene Solution (Gibco, Cat# 15040066) every 3-5 days. RevitaCell^{TM} supplement (Thermo Fisher, Cat# A2644501) was added for 24 hrs after passaging or thawing. Primed human ES cells were cultured in a humidified incubator at 37°C and 5% CO_2. The human 293T cells was purchased

from The American Type Culture Collection (ATCC, Cat# CRL3216). Human 293T cells were cultured in DEME medium (Thermo Fisher, Cat# C11995500BT) supplemented with 10% Fetal Bovine Serum (VisTech, Cat# SE100-011) and 1% Penicillin-Streptomycin (Thermo Fisher, Cat# 15140122) in a humidified incubator at 37°C and 5% CO_2. Cells were passaged using TrypLE™ Express Enzyme (Thermo Fisher, Cat# 12604021) every 2-3 days.

METHOD DETAILS

Construction of the WGS libraries

Genomic DNA (gDNA) was purified from blood samples of human oocyte donors or semen sample of human sperm donors by using DNeasy Blood & Tissue Kits (Qiagen, Cat# 69504) according to the manufacturer's protocol. Then, gDNA samples were sheared to 300 bp by using the Covaris M220 instrument. Next, fragmented gDNA was processed to construct WGS library by using the NEBNext UltraII DNA Library Prep Kit (New England Biolabs, Cat# E7645L) according to the manufacturer's protocol. Finally, libraries were sequenced on an Illumina NovaSeq 6000 sequencer with a 150 bp paired-end sequencing strategy.

QUANTIFICATION AND STATISTICAL ANALYSIS

Identifying germline SNPs using whole-genome sequencing

To accurately identify allele-specific alignments from sequencing data, whole-genome sequencing was performed on peripheral blood or sperm from donors to identify parental single-nucleotide polymorphisms (SNPs). Sequencing reads were aligned to the reference genome sequences (UCSC version hg19) using bwa (v0.7.12). The GATK (v4.2.6.1) germline short variant discovery pipeline was further applied to identify SNPs. Raw SNPs were filtered by *VariantFiltration* in GATK with the following parameters: *QD < 2.0* || *FS > 60.0* || *MQ < 40.0* || *MQRankSum < -12.5* || *ReadPosRankSum* < *-8.0*. Only homozygous SNPs in donors were used for identifying allele-specific alignments from sequencing data.

Identifying allele-specific alignments from sequencing data

The allele-specific alignments were identified using SNPsplit (v0.5.0). Briefly, the N-masked hg19 human genome was generated based on parental SNPs. The trimmed reads were aligned to the N-masked hg19 human genome as described above. The aligned reads were further grouped into maternal or paternal reads using SNPsplit for further analysis.

Identifying maintenance methylated regions

To identify regions with maintenance high DNA methylation, tiles with differences of less than 0.15 in methylation levels between two groups and methylation levels higher than 0.65 in both groups were retained. These regions were further merged if they were separated by less than 10 kb. Regions that covered at least nine CpG sites in the aggregated data and had no significant differences (FDR-adjusted multiple t-test with adjusted *P*-values higher than 0.05) were defined as maintenance high methylation regions. Oocyte hyper hmDMRs were classified into three distinct categories based on the 5hmC levels between oocytes and the maternal genome of zygotes. The categorization was as follows: Group C, characterized by a pronounced loss of 5hmC, where the level of 5hmC in the maternal genome of the zygote was less than 10% of the average level observed in the oocyte; Group B, denoted as partial loss, with 5hmC levels in the maternal genome of the zygote falling below 50% of the oocyte's average; and Group A, termed maintenance, which comprised the remaining regions not fitting the criteria for Groups B or C. Regions where the DNA methylation level decreases by more than 25% post fertilization were considered as demethylated regions. Similarly, regions with an increase in methylation level by more than 25% were considered as *de novo* methylated regions. And regions where the change in methylation levels is within ±25 %, indicating a stable methylation state, were considered as methylation maintenance regions.

Quantifying the expression in RNA-seq data

Genes down-regulated by the small molecule inhibitors DMOG and Bobcat339 were characterized by the following criteria: baseline expression levels exceeding 0.5 in the DMSO control group, and expressed in both the DMOG and Bobcat339 groups. Additionally, these genes were with an average expression level greater than 0.1 at the 4-cell and 8-cell stages. Genes were also required to be up-regulated in the 4-cell or 8-cell stages relative to the MII oocyte stage, with a minimum $\log_2$ fold change of 1, and down-regulated in the presence of inhibitors, with a minimum $\log_2$ fold change of 0.5.

Genes activated during the 4-8 cell stages are characterized by specific expression thresholds: an average expression level below 1 at the MII oocyte stage, equal to or below 1 at the preceding stage (either 2-cell or 4-cell), and above 1 at the current stage (either 4-cell or 8-cell). Furthermore, the expression levels at the 4-cell or 8-cell stages must be higher than those at the MII oocyte stage, with a fold-change minimum of 6.

To define genes with ectopic hyper hmDMRs, these hmDMRs in mTet3-overexpressing (OE) human 4-cell embryos that overlapped with known human enhancers were identified. The genes closest to these identified regions were tagged as having ectopic hyper hmDMRs. These genes were further evaluated between the control and OE groups. For RNA expression levels, only those showing a minimum log2 fold change of 1.5 were considered. Additionally, the ratio of 5hmC to 5mC for hmDMRs linked to these genes was compared between the control and OE groups, setting a threshold minimum log2 fold change of 2. Genes with a log2 fold change in RNA expression greater than 1.5 and a log2 fold change in the 5hmC/5mC ratio above 2 were categorized as activated genes in mTet3-plus OE. Similarly, genes with a log2 fold change in RNA expression less than -1.5 and a log2 fold change in the 5hmC/5mC ratio above 2 were categorized as down-regulated genes. The others were considered as insensitive genes.

Annotations of genomic regions

Annotations for CpG islands, exons, introns, transcriptional start sites (TSSs),

transcriptional end sites (TESs), and repetitive elements were retrieved from the UCSC Genome Browser (UCSC version mm9 and hg19). The gene body was defined as the region between the TSS and TES. Intergenic regions were defined as the regions outside of the gene body. The promoter was the region flanking the TSS (1,000 bp upstream and 500 bp downstream). Annotations for imprinted germline DMRs and enhancers in mm9 and hg19 were retrieved from previous studies. For regions in Figure 4, histone modification peaks were retrieved from previous studies. Promoters with H3K4me3 and H3K27me3 peaks were considered bivalent promoters. H3K27me3 peaks in 2 kb regions flanking the TSS were considered proximal peaks, and the others were considered distal peaks. H3K9me3 peaks overlapping with NDRs were considered euchromatin. H3K9me3 peaks with GCH levels less than the median GCH level in H3K9me3 peaks were considered heterochromatin. UCSC LiftOver was used to convert genome coordinates between human hg19 and mouse mm9.

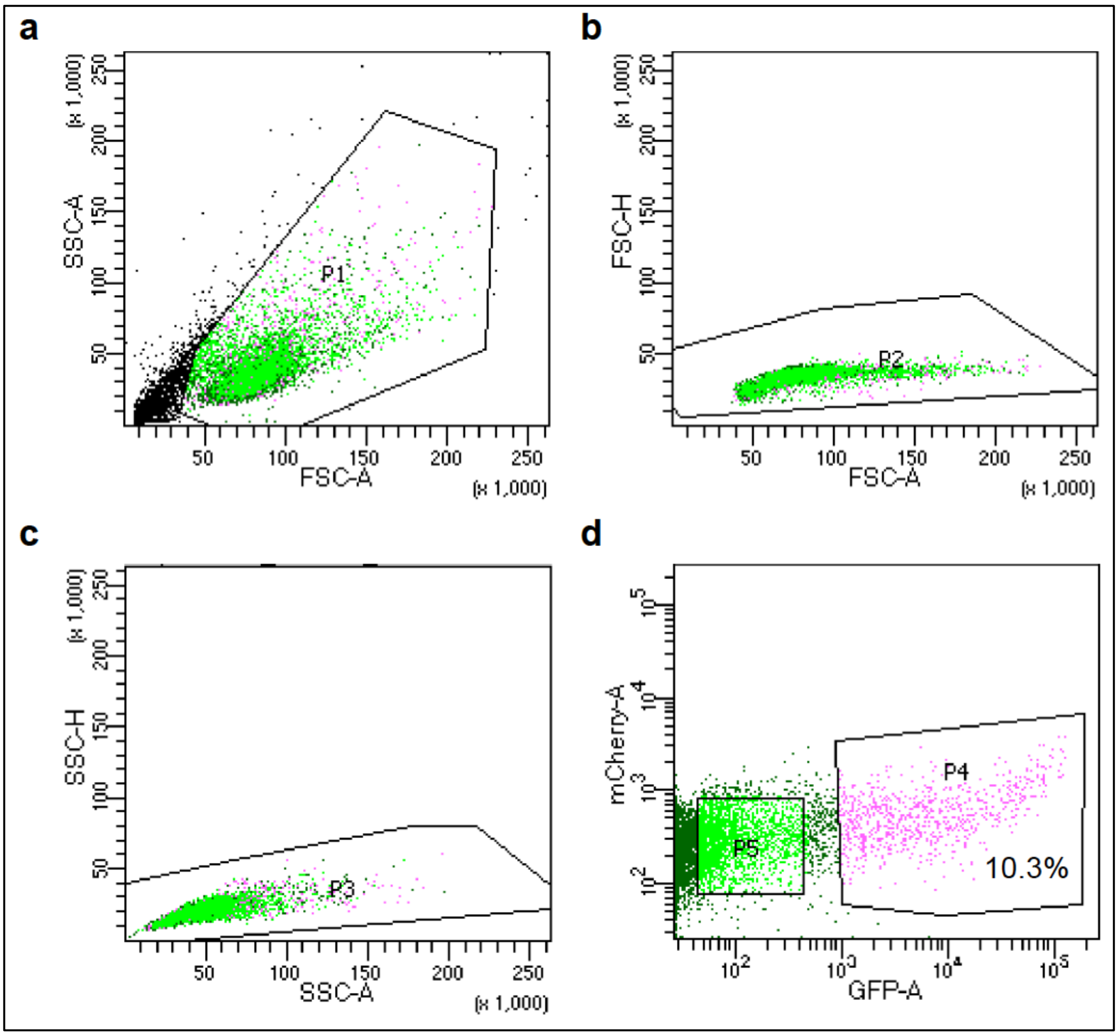

Supplementary Figure 1. Gating strategy for fluorescence-activated cell sorting (FACS) of EGFP positive cells. FSC-A × SSC-A were used to excludes debris. Then, FSC-A × FSC-H and SSC-A × SSC-H were used to remove doublets and clumps. Positive and negative boundaries were determined by control cells.

STATISTICS SOURCE DATA TABLE

**Figure 4a**		
		Two-sided Wilcoxon signed-rank test
H3K4me3 only and Bivalent	Oocyte	p < 2.22e−16
	4-cell	p = 0.24
	8-cell	p = 0.0037
	Blastocyst	p = 0.000000000096
	hESC	p < 2.22e−16
Proximal and Distal H3K27me3	Oocyte	p < 2.22e−16
	4-cell	p = 0.0000092
	8-cell	p = 0.32
	Blastocyst	p < 2.22e−16
	hESC	p = 0.21
H3K9me3 in euchromatin and heterochromatin	4-cell	p < 2.22e−16
	8-cell	p < 2.22e−16
	Blastocyst	p < 2.22e−16
	hESC	p < 2.22e−16
H3K27ac w/ NDR and w/o NDR	8-cell	p < 2.22e−16
	Blastocyst	p < 2.22e−16
	hESC	p < 2.22e−16
**Figure 5e**		
		Two-sided Wilcoxon signed-rank test
Control and OE mouse enhanced Tet3	5mC	p < 2.22e−16
	5hmC	p < 2.22e−16
**Figure 6e**		
		Two-sided Student's t-test
Control and OTX2 KD	*OTX2*	p = 0.0013
**Extended Data Figure 6b**		
		Two-sided Wilcoxon signed-rank test

ZGA and Random genes (Promoter)	Oocyte	p = 0.238
	Zygote	p = 0.681
	2-cell	p = 0.079
	4-cell	p = 0.498
	8-cell	p = 0.098
	Blastocyst	p = 0.447
	hESC	p = 0.013
ZGA and Random genes (Genebody)	Oocyte	p = 0.0000000028
	Zygote	p = 0.0002
	2-cell	p = 0.00738
	4-cell	p = 0.00442
	8-cell	p = 0.00071
	Blastocyst	p = 0.0000000023
	hESC	p = 0.01237
**Extended Data Figure 8a**		
		Two-sided Student's t-test
Control and OE mouse enhanced Tet3	*mTet3-plus*	p = 0.02641
	ZSCAN4	p = 0.4828
	LEUTX	p = 0.8138
	DUXB	p = 0.347
	ZNF280A	p = 0.4897
	ACTB	p = 0.6223
**Extended Data Figure 8b**		
		Two-sided Wilcoxon signed-rank test
Control and OE mouse enhanced Tet3	Genome-wide	p < 2.22e−16
	Gene body	p < 2.22e−16
	Promoter	p < 2.22e−16
	Intergenic	p < 2.22e−16
	CGI	p = 0.0014

	SINE	p < 2.22e−16
	LINE	p < 2.22e−16
	LTR	p < 2.22e−16
	SVA	p < 2.22e−16
**Extended Data Figure 8c**		
		Two-sided Wilcoxon signed-rank test
Control and OE mouse enhanced Tet3	Genome-wide	p < 2.22e−16
	Gene body	p < 2.22e−16
	Promoter	p < 2.22e−16
	Intergenic	p < 2.22e−16
	CGI	p < 2.22e−16
	SINE	p < 2.22e−16
	LINE	p < 2.22e−16
	LTR	p < 2.22e−16
	SVA	p < 2.22e−16
**Extended Data Figure 8f**		
		Two-sided Wilcoxon signed-rank test
Control and OE mouse enhanced Tet3	5hmC	p = 0.0000000009961
	5mC	p < 2.22e−16
**Extended Data Figure 8k**		
		Two-sided Wilcoxon signed-rank test
Activated and Insensitive genes	Oocyte	p =0.0000000000048
	Zygote	p = 0.0000000004
	2-cell	p = 0.000000000029
	4-cell	p = 0.000000000033
	8-cell	p = 0.00000000062
	Blastocyst	p = 0.000025
	hESC	p = 0.000012
**Extended Data Figure 8l**		

		Two-sided Wilcoxon signed-rank test
Control and OE mouse enhanced Tet3	Activated genes	p < 2.22e−16
**Extended Data Figure 9d**		
		Two-sided Wilcoxon signed-rank test
Control and OTX2 KD	*OTX2*	p = 0.0000068
	ESRRB	p = 0.028
	DUSP4	p = 0.016
**Extended Data Figure 9e**		
		Two-sided Student's t-test
Control and OE OTX2	*OTX2*	p = 0.0008

SUPPLEMENTARY TABLE LEGENDS

Supplementary Table 1: Statistics of whole-genome DNA hydroxymethylation sequencing data information, related to Figure 1.

Supplementary Table 2: DNA hydroxymethylation information at maternal germline differentially methylated regions (mgDMRs) in human and mouse gametes, related to Figure 1d.

Supplementary Table 3: Hyper hmDMRs in human oocyte (n=20,763), related to Figure 2.

Supplementary Table 4: Hyper hmDMRs in human male pronucleus (n=1,201), related to Figure 3.

Supplementary Table 5: Expression level of 5hmC-sensitive embryonic genes (n=691), related to Figure 4.

Supplementary Table 6: Hypo DMRs in mTet3-plus overexpressed human 4-cell embryos (n=20,594), related to Figure 5.

Supplementary Table 7: Hyper hmDMRs in mTet3-plus overexpressed human 4-cell embryos (n=19,920), related to Figure 5.

Supplementary Table 8: Expression level of activated genes with ectopically generated hyper hmDMRs (n=342), related to Figure 5.

Supplementary Table 9: Expression level of OTX2 targeted genes (n=999), related to Figure 6.
