# Supplementary material for: Distinct dynamics of parental 5-hydroxymethylcytosine during human preimplantation development regulate early lineage gene expression
Source: Nat Cell Biol. 2024 Jul 30;26(9):1458–69. doi: 10.1038/s41556-024-01475-y (PMC11392820; doi:10.1038/s41556-024-01475-y)
Supplement: Supplementary file 2 — Reporting Summary [file 41556_2024_1475_MOESM2_ESM.pdf]

nature portfolio

Corresponding author(s): Fan Guo, Dan Liang, Yunxia Cao, Xiaojin He

Last updated by author(s): Jun 19, 2024

Reporting Summary

Nature Portfolio wishes to improve the reproducibility of the work that we publish. This form provides structure for consistency and transparency in reporting. For further information on Nature Portfolio policies, see our Editorial Policies and the Editorial Policy Checklist.

Statistics

For all statistical analyses, confirm that the following items are present in the figure legend, table legend, main text, or Methods section.

n/a	Confirmed	
☐	☒	The exact sample size (n) for each experimental group/condition, given as a discrete number and unit of measurement
☐	☒	A statement on whether measurements were taken from distinct samples or whether the same sample was measured repeatedly
☐	☒	The statistical test(s) used AND whether they are one- or two-sided<br>*Only common tests should be described solely by name; describe more complex techniques in the Methods section.*
☒	☐	A description of all covariates tested
☒	☐	A description of any assumptions or corrections, such as tests of normality and adjustment for multiple comparisons
☐	☒	A full description of the statistical parameters including central tendency (e.g. means) or other basic estimates (e.g. regression coefficient) AND variation (e.g. standard deviation) or associated estimates of uncertainty (e.g. confidence intervals)
☐	☒	For null hypothesis testing, the test statistic (e.g. F, t, r) with confidence intervals, effect sizes, degrees of freedom and P value noted<br>*Give P values as exact values whenever suitable.*
☒	☐	For Bayesian analysis, information on the choice of priors and Markov chain Monte Carlo settings
☒	☐	For hierarchical and complex designs, identification of the appropriate level for tests and full reporting of outcomes
☐	☒	Estimates of effect sizes (e.g. Cohen's d, Pearson's r), indicating how they were calculated

Our web collection on statistics for biologists contains articles on many of the points above.

Software and code

Policy information about availability of computer code

Data collection	ZEN (2012 SP2), BD FACSDiva 9.0, Compass for SW v4.1.0, illumina Novaseq 6000
Data analysis	TrimGalore (v0.6.6), Bismark (v0.23.1), sambamba (v0.8.2), MethylDackel (v0.5.0), bwa (v0.7.12), GATK (v4.2.6.1), SNPsplit (v0.5.0), MLML (v.5.0.0), samtools (v1.17), SEACR (v1.3), STAR (v2.7.10b), featureCounts (v2.0.4), HOMER (v4.11), MOODS (v1.9.4), UCSC LiftOver

For manuscripts utilizing custom algorithms or software that are central to the research but not yet described in published literature, software must be made available to editors and reviewers. We strongly encourage code deposition in a community repository (e.g. GitHub). See the Nature Portfolio guidelines for submitting code & software for further information.

Data

Policy information about availability of data

All manuscripts must include a data availability statement. This statement should provide the following information, where applicable:

- Accession codes, unique identifiers, or web links for publicly available datasets
- A description of any restrictions on data availability
- For clinical datasets or third party data, please ensure that the statement adheres to our policy

Sequencing data that support the findings of this study have been deposited in the Gene Expression Omnibus (GEO) under accession code GSE224618, and GSA (HRA006264) and OMIX (OMIX005397 and OMIX005398) in the National Genomics Data Center (NGDC, https://bigd.big.ac.cn/). The Human reference genome hg19 was obtained from UCSC (https://hgdownload.cse.ucsc.edu/goldenpath/hg19/chromosomes/). Previously published datasets that were re-analysed here are

available under the following accession codes: ACE-seq data of mouse gametes and preimplantation embryos (GSE186357), scRNA-seq data of human oocytes, preimplantation embryos and hESCs (GSE36552), Ribo-RNA-lite data of human mature oocytes, preimplantation embryos and hESCs (GSE197265), scBS-seq data of human gametes and preimplantation embryos (GSE81233), and scCOOL-seq data of human gametes, preimplantation embryos and ESCs (GSE100272). scChaRM-seq data of human growing oocytes and mature oocytes (GSE154762). Human ESCs, oocytes and early embryonic dataset with peaks of H3K4me3, H3K27me3, H3K27ac and H3K9me3 are deposited as GSE124718, GSE176016 and GSE52824. Proteome data of human preimplantation embryos is available via the integrated proteome resources (iProX) of ProteomeXchange (PXD024267). Source data are provided with this study. All other data supporting the findings of this study are available from the corresponding author on reasonable request.

Research involving human participants, their data, or biological material

Policy information about studies with human participants or human data. See also policy information about sex, gender (identity/presentation), and sexual orientation and race, ethnicity and racism.

Reporting on sex and gender	We recruited male donors to provide sperm and female donors to provide oocytes, and used ICSI to obtain early human embryos. Gender was not relevant in this study.
Reporting on race, ethnicity, or other socially relevant groupings	This study is not related to race, ethnicity or other relevant social groups.
Population characteristics	Thirty-two oocyte donors, aged 21-40 years, were recruited from females undergoing regular IVF treatments. These patients generously donated surplus eggs to support our scientific research, with no additional hormonal stimulation or egg retrieval performed solely for donation purposes. Two healthy sperm donors, aged 23 and 36 years respectively.
Recruitment	Two healthy sperm donors were recruited locally through print advertisements. Fresh mature oocytes and immature oocytes were voluntarily donated from females undergoing regular IVF treatment.
Ethics oversight	This research was designed to study the dynamics and regulatory mechanisms of DNA hydroxymethylation in human preimplantation embryos and germ cells. The study analyzed the DNA hydroxymethylation dynamics in sperm, MII oocytes, zygotes, 2-cell, 4-cell, 8-cell embryos, blastocysts and human embryonic stem cells (hESCs). Besides, 3PN embryos were collected for functional verification experiments by using RNA-seq, ACE-seq, WGBS and immunofluorescence. The embryos were cultured in vitro for a maximum of six days. All the experimental procedures mentioned above have been reviewed and approved by the Biomedical Ethics Committee of Anhui Medical University (83220416). The First Affiliated Hospital of Anhui Medical University was responsible for recruiting oocyte and sperm donors for the study. Written informed consent was obtained from all donors prior to enrolling in the study. Before signing informed consent, persons donating germ cells were provided with all the necessary information, including an introduction to this research, usage of donated samples, protection of privacy, a means to receive counselling, as well as the risk, gain and right of participation. Additionally, an opportunity for refusal to participate in research was guaranteed by an opt-out manner. This research was conducted ethically in accordance with the measures of the People's Republic of China on the administration of Human Assisted Reproductive Technology, the ethical principles of the Human Assisted Reproductive Technology and the Human Sperm Bank as well as the Declaration of Helsinki. Experiments on human early embryos and hESCs also followed the 2016 Standards for Human Stem Cell Use in Research issued by the International Society for Stem Cell Research (ISSCR).

Note that full information on the approval of the study protocol must also be provided in the manuscript.

Field-specific reporting

Please select the one below that is the best fit for your research. If you are not sure, read the appropriate sections before making your selection.

☒ Life sciences ☐ Behavioural & social sciences ☐ Ecological, evolutionary & environmental sciences

For a reference copy of the document with all sections, see nature.com/documents/nr-reporting-summary-flat.pdf

Life sciences study design

All studies must disclose on these points even when the disclosure is negative.

Sample size	No statistical methods were used to predetermine the sample size, but our sample sizes are similar to those reported in previous publications (Yan et al. Nature Genetics 2023, Zou et al. Science 2022, Gassler et al. Science 2022).
Data exclusions	No early human embryo samples or data points were excluded from the analyses for any reason.
Replication	For ACE-seq of 2PN human early embryos, the number of biological replicates for each sample was: Sperm, n=2; Oocyte, n=4; Zygote, n=3; 2-cell, n=3; 4-cell, n=4; 8-cell, n=5; blastocyst, n=3; hESC, n=1. For RNA-seq of control and OTX2 overexpressed human ES cells, n=2 and 3, respectively. For RNA-seq of negative control and OTX2 knockdown human day 4 embryos, n=12 and 9 respectively. For RNA-seq of DMSO, DMOG and Bobcat339 treated human day 4 embryos, n=21, 16 and 17, respectively. For RNA-seq of control and mouse enhanced Tet3 overexpressed human 3PN 8-cell embryos, n=2, respectively. For WGBS of human embryonic stem cells, n=2. For CUT&Tag of control and OTX2 overexpressed human embryonic stem cells, n=1 and 2, respectively. For qPCR to validate the successful knockdown of OTX2 in human day 4 embryos, n=4 for negative control embryos, n=9 for OTX2 KD embryos. For qPCR to validate the successful overexpression of OTX2 in hESCs, n=3 was chosen as the minimal replicate number. For imaging experiments, 2-9 biological replicates or embryos were performed, the exact number is indicated in the respective figure legend. For western blot, two independent experiments were performed. All attempts at

replication were successful.

Randomization	In all the experiment included in this study, human early embryos, hESCs and h293T cells were collected and randomly allocated to control and treatment group without a preconceived selection strategy or prioritization by morphology or state.
Blinding	The investigators were not blinded to allocation during experiments and outcome assessment. Based on previous studies in this field, blinding was not relevant for this study since this is not an intervention study. Whether sequencing data or biochemical experiments, our analytical pipeline or parameters for image data collection followed uniform criteria applied to all samples, allowing us to analyse our data in an unbiased manner.

Reporting for specific materials, systems and methods

We require information from authors about some types of materials, experimental systems and methods used in many studies. Here, indicate whether each material, system or method listed is relevant to your study. If you are not sure if a list item applies to your research, read the appropriate section before selecting a response.

Materials & experimental systems

n/a	Involved in the study
☐	☒ Antibodies
☐	☒ Eukaryotic cell lines
☒	☐ Palaeontology and archaeology
☒	☐ Animals and other organisms
☒	☐ Clinical data
☒	☐ Dual use research of concern
☒	☐ Plants

Methods

n/a	Involved in the study
☐	☒ ChIP-seq
☐	☒ Flow cytometry
☒	☐ MRI-based neuroimaging

Antibodies

Antibodies used

Rabbit monoclonal anti-GAPDH, Cell Signaling Technology, Cat# 2118s, Clone# 14C10, for WES (1:100 duiltion) and Immunobloting (1:1000 dilution)
Mouse monoclonal anti-FLAG, Sigma Cat# F1804, Clone# M2, for WES (1:20 dilution) and immunobloting (1:2000 dilution)
Rabbit polyclonal anti-FLAG, Sigma, Cat# F7425, for Cut&Tag (4ug) and immunofluorescence (1:500 dilution)
Mouse monoclonal anti-OTX2, Santa Cruz, Cat# sc-514195, Clone# D-8, for immunofluorescence (1:200 dilution)
Mouse monoclonal anti-HA, Abcam, Cat# ab18181, Clone# HA.C5, for immunofluorescence (1:500 dilution)
Mouse monoclonal anti-HA, Abclonal, Cat# AE008, Clone# AMC0503, for immunobloting (1:2000 dilution)
Mouse monoclonal anti-HRP-conjugated beta actin, Proteintech, Cat# HRP-60008, Clone# 7D2C10, for immunobloting (1:2000 dilution)
Rabbit polyclonal anti-5hmC, Active motif, Cat# 39791, for immunofluorescence (1:500 dilution)
Mouse monoclonal anti-5mC, Diagenode, Cat# C15200081-100, Clone# 33DC, for immunofluorescence (1:500 dilution)
Goat polyclonal anti-rabbit IgG, Abcam, Cat# ab6702, for Cut&Tag (1:100 dilution)
Goat anti-mouse IgG, Horseradish Peroxidase-conjugated, Proteintech, Cat# SA00001-1, for Immunobloting (1:1000 dilution)
Goat anti-rabbit IgG, Horseradish Peroxidase-conjugated, Proteintech, Cat# SA00001-2, for Immunobloting (1:1000 dilution)
Goat polyclonal anti-Mouse Alexa Fluor® 488, Invitrogen, Cat# A11029, for immunofluorescence (1:1000 dilution)
Goat polyclonal anti-Rabbit Alexa Fluor® 555, Invitrogen, Cat# A21429, for immunofluorescence (1:1000 dilution)

Validation

All the antibodies used in this study are commercially available and have validation statements on the manufacturer's website:
Rabbit monoclonal anti-GAPDH: https://www.cellsignal.com/products/primary-antibodies/gapdh-14c10-rabbit-mab/2118
Mouse monoclonal anti-FLAG: https://www.sigmaaldrich.cn/CN/zh/product/sigma/f1804
Rabbit polyclonal anti-FLAG: https://www.sigmaaldrich.cn/CN/zh/product/sigma/f7425?
Mouse monoclonal anti-OTX2: https://www.scbt.com/p/otx2-antibody-d-8
Mouse monoclonal anti-HA: https://www.abcam.com/products/primary-antibodies/ha-tag-antibody-hac5-ab18181.html
Mouse monoclonal anti-HA: https://abclonal.com.cn/catalog/AE008
Mouse monoclonal anti-HRP-conjugated beta actin: https://www.ptglab.com/products/ACTB-Antibody-HRP-60008.htm
Rabbit polyclonal anti-5hmC: https://www.activemotif.com/catalog/details/39791/5-hydroxymethylcytidine-antibody-pab
Mouse monoclonal anti-5mC: https://www.diagenode.com/en/p/5-mc-monoclonal-antibody-33d3-premium-100-ug-50-ul
Goat polyclonal anti-rabbit IgG: https://www.abcam.com/products/secondary-antibodies/goat-rabbit-igg-hl-ab6702.html
Goat anti-mouse IgG, Horseradish Peroxidase-conjugated: https://www.ptglab.com/products/HRP-conjugated-Affinipure-Goat-Anti-Mouse-IgG-H-L-secondary-antibody.htm
Goat anti-rabbit IgG, Horseradish Peroxidase-conjugated: https://www.ptglab.com/products/HRP-conjugated-Affinipure-Goat-Anti-Rabbit-IgG-H-L-secondary-antibody.htm
Goat polyclonal anti-Mouse Alexa Fluor® 488: https://www.thermofisher.cn/cn/zh/antibody/product/Goat-anti-Mouse-IgG-H-L-Highly-Cross-Adsorbed-Secondary-Antibody-Polyclonal/A-11029
Goat polyclonal anti-Rabbit Alexa Fluor® 555: https://www.thermofisher.cn/cn/zh/antibody/product/Goat-anti-Rabbit-IgG-H-L-Highly-Cross-Adsorbed-Secondary-Antibody-Polyclonal/A-21429

Eukaryotic cell lines

Policy information about cell lines and Sex and Gender in Research

Cell line source(s)	The primed human ES cell line (WIBR3) was a gift from Professor Hao-Yi Wang (State Key Laboratory of Stem Cell and Reproductive Biology, Institute of Zoology, Chinese Academy of Sciences, Beijing 100101, China). The human 293T cells was purchased from The American Type Culture Collection (ATCC, Cat# CRL3216).
Authentication	Human 293T cell line was authenticated by The American Type Culture Collection (STR profiling).
Mycoplasma contamination	Cell lines were routinely tested for mycoplasma contamination by PCR and confirmed that they were negative for mycoplasma contamination.
Commonly misidentified lines (See ICLAC register)	None of the cell lines used was listed in the database of ICLAC.

Plants

Seed stocks	*Report on the source of all seed stocks or other plant material used. If applicable, state the seed stock centre and catalogue number. If plant specimens were collected from the field, describe the collection location, date and sampling procedures.*
Novel plant genotypes	*Describe the methods by which all novel plant genotypes were produced. This includes those generated by transgenic approaches, gene editing, chemical/radiation-based mutagenesis and hybridization. For transgenic lines, describe the transformation method, the number of independent lines analyzed and the generation upon which experiments were performed. For gene-edited lines, describe the editor used, the endogenous sequence targeted for editing, the targeting guide RNA sequence (if applicable) and how the editor was applied.*
Authentication	*Describe any authentication procedures for each seed stock used or novel genotype generated. Describe any experiments used to assess the effect of a mutation and, where applicable, how potential secondary effects (e.g. second site T-DNA insertions, mosiacism, off-target gene editing) were examined.*

ChIP-seq

Data deposition

☒ Confirm that both raw and final processed data have been deposited in a public database such as GEO.

☒ Confirm that you have deposited or provided access to graph files (e.g. BED files) for the called peaks.

Data access links *May remain private before publication.*	All raw sequencing data and the corresponding processed files generated of CUT&Tag were deposited to NCBI GEO database (GSE224618).
Files in database submission	CUTTag_hESC_control_input_RPKM_bin50.bw; CUTTag_hESC_OE_OTX2_Rep1_RPKM_bin50.bw; CUTTag_hESC_OE_OTX2_Rep2_RPKM_bin50.bw; CUTTag_hESC_OE_OTX2.cleanPeaks.bed
Genome browser session (e.g. UCSC)	NA

Methodology

Replicates	2 replicate for Flag CUT&Tag in OTX2 overexpressed hESCs; 1 replicate for Flag CUT&Tag in control hESCs.
Sequencing depth	5G/sample
Antibodies	Rabbit polyclonal anti-FLAG, Sigma, Cat# F7425, for Cut&Tag (4ug)<br>Goat polyclonal anti-rabbit IgG, Abcam, Cat# ab6702, for Cut&Tag (1:100 dilution)
Peak calling parameters	Peak calling was performed using SEACR (v1.3).
Data quality	Adaptors were trimmed from raw sequences using Trim Galore (v0.6.6). The trimmed reads were aligned to the human reference genome (hg19) using bowtie2 with parameters: --local --verysensitive --no-mixed --no-discordant --phred33 -I 10 -X 700. PCR duplicates were identified and removed using sambamba (v0.8.2). Unmapped reads were further excluded using samtools (v1.17). Read pairs that were on the same chromosome and fragment length less than 1000 bp were used for the downstream analysis. The enriched regions in OTX2-overexpressed hESC were called using the wildtype hESC as control track.
Software	Trim Galore (v0.6.6), sambamba (v0.8.2), samtools (v1.17), SEACR (v1.3).

Flow Cytometry

Plots

Confirm that:

☒ The axis labels state the marker and fluorochrome used (e.g. CD4-FITC).

☒ The axis scales are clearly visible. Include numbers along axes only for bottom left plot of group (a 'group' is an analysis of identical markers).

☒ All plots are contour plots with outliers or pseudocolor plots.

☒ A numerical value for number of cells or percentage (with statistics) is provided.

Methodology

Sample preparation	Human ES cells were cultured in Essential-8 medium (Thermo Fisher, Cat# A1517001) containing Matrigel (Corning, Cat# 354277). Cells were passaged using Versene Solution (Gibco, Cat# 15040066) every 3-5 days. RevitaCell supplement (Thermo Fisher, Cat# A2644501) was added for 24 hrs after passaging or thawing. Primed human ES cells were cultured in a humidified incubator at 37°C and 5% CO_2. To overexpress OTX2 in human ES cells, plasmids were transiently transfected into human ES cells by using Lipofectamine™ Stem Transfection Reagent (Thermo Fisher, Cat# STEM00003). After transfection for 24 hrs, the EGFP signal was observed in overexpressed human ES cells under a fluorescence microscope. Then, the cells were digested with Versene Solution and prepared for FACS. EGFP-positive cells were sorted and kept in DPBS supplemented with 0.04% BSA and ROCK inhibitor (10 µM Y-27632, Selleck, Cat# s1049) for western blot analysis and RNA-seq.
Instrument	BD FACS Fusion flow cytometer was used for data acquisition.
Software	BD FACSDiva 9.0 software was used for data collection and analysis.
Cell population abundance	Cell population abundance was influenced by the transfection efficiency in hESCs. The purity of sample was determined by the EGFP-positive signal in each cells under a fluorescence microscope after sorting.
Gating strategy	Firstly, FSC-A × SSC-A were used to excludes debris. Then, FSC-A × FSC-H and SSC-A × SSC-H were used to remove doublets and clumps. Positive and negative boundaries were determined by control cells.

☒ Tick this box to confirm that a figure exemplifying the gating strategy is provided in the Supplementary Information.
