# Supplementary figures and images for: Distinct dynamics of parental 5-hydroxymethylcytosine during human preimplantation development regulate early lineage gene expression
Source: Nat Cell Biol. 2024 Jul 30;26(9):1458–69. doi: 10.1038/s41556-024-01475-y (PMC11392820; doi:10.1038/s41556-024-01475-y)

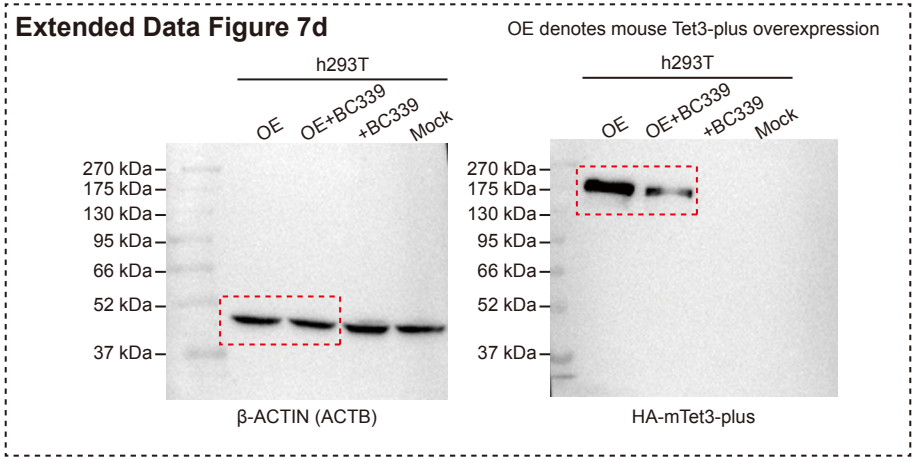

Supplement: Supplementary file 4 — Unprocessed western blots of Extended Data Fig. 7. [file 41556_2024_1475_MOESM4_ESM.pdf]

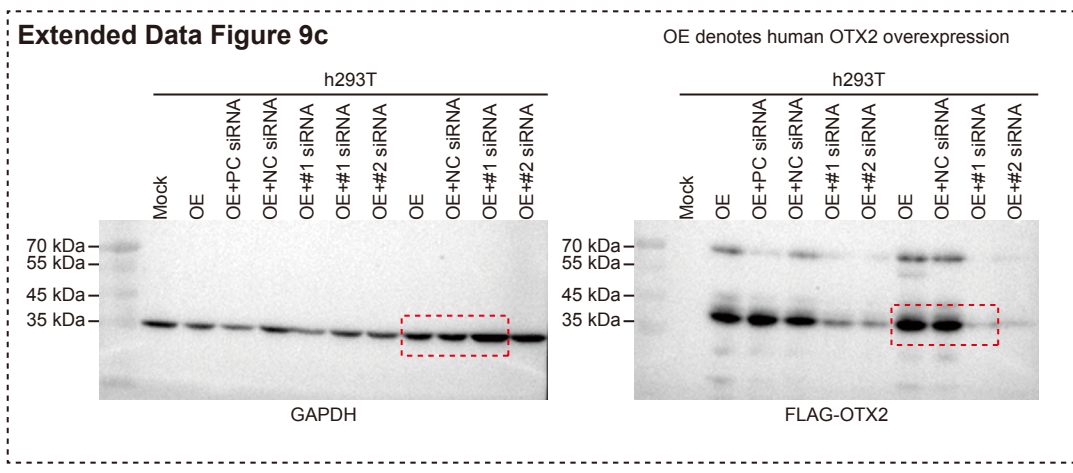

Supplement: Supplementary file 5 — Unprocessed western blots of Extended Data Fig. 9. [file 41556_2024_1475_MOESM5_ESM.pdf]
